# Landscape structure, climate variability, and soil quality shape crop biomass patterns in agricultural ecosystems of Bavaria

**DOI:** 10.3389/fpls.2025.1630087

**Published:** 2025-08-07

**Authors:** Maninder Singh Dhillon, Thomas Koellner, Sarah Asam, Jakob Bogenreuther, Stefan Dech, Ursula Gessner, Daniel Gruschwitz, Sylvia Helena Annuth, Tanja Kraus, Thomas Rummler, Christian Schaefer, Sarah Schönbrodt-Stitt, Ingolf Steffan-Dewenter, Martina Wilde, Tobias Ullmann

**Affiliations:** ^1^ Department of Remote Sensing, Institute of Geography and Geology, University of Würzburg, Würzburg, Germany; ^2^ Department of Ecological Services, Faculty of Biology, Chemistry and Earth Sciences, Bayreuth Center of Ecology and Environmental Research (BayCEER), University of Bayreuth, Bayreuth, Germany; ^3^ German Remote Sensing Data Center (DFD), German Aerospace Center (Deutsches Zentrum für Luft- und Raumfahrt (DLR), Wessling, Germany; ^4^ Department of Applied Computer Science, Institute of Geography, University of Augsburg, Augsburg, Germany; ^5^ Department of Animal Ecology and Tropical Biology, University of Würzburg, Würzburg, Germany; ^6^ Department of Physical Geography and Soil Science, Institute of Geography and Geology, University of Würzburg, Würzburg, Germany

**Keywords:** crop biomass modeling, landscape diversity, climate variability, random forest regression, small woody features, climate-resilient agriculture, sustainable agriculture, machine learning

## Abstract

Understanding how environmental variability shapes crop biomass is essential for improving yield stability and guiding climate-resilient agriculture. To address this, we compared biomass estimates from a semi-empirical light use efficiency (LUE) model with predictions from a machine learning–remote sensing framework that integrates environmental variables. We applied a combined LUE and random forest (RF) model to estimate the mean biomass of winter wheat and oilseed rape across Bavaria, Germany, from 2001 to 2019. Using a 5 km2 hexagon-based grid, we incorporated landscape metrics (land cover diversity, small woody features), topographic variables (elevation, slope, aspect), soil potential, and seasonal climate predictors (mean and standard deviation of temperature, precipitation, and solar radiation) across the growing season. The RF-based approach improved predictive accuracy over the LUE model alone, particularly for winter wheat. Biomass patterns were shaped by both landscape configuration and climatic conditions. Winter wheat biomass was more influenced by topographic and landscape features, while oilseed rape was more sensitive to solar radiation and soil properties. Moderately diverse landscapes supported higher biomass, whereas an extreme landscape fragmentation or high variability showed lower values. Temperature thresholds, above 21 °C for winter wheat and 12 °C for oilseed rape, were associated with biomass declines, indicating crop-specific sensitivities under Bavarian conditions. This hybrid modeling approach provides a transferable framework to map and understand crop biomass dynamics at scale. The findings offer region-specific insights that can support sustainable agricultural planning in the context of climate change.

## Graphical Abstract

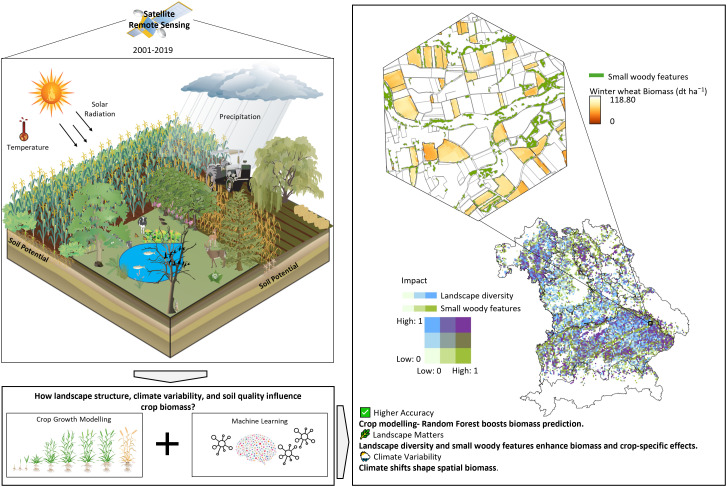

## 1 Introduction

Understanding the spatial and temporal drivers of crop productivity is essential for enhancing agricultural resilience in the realm of climate change, land-use intensification, and biodiversity loss (Foley et al., 2011; Kastner et al., 2022; Ray et al., 2015). Across temperate Europe, rising temperatures, shifting precipitation regimes, and land-use changes have altered the distribution of yields for major crops such as winter wheat (WW) and oilseed rape (OSR) (Bönecke et al., 2020; Faye et al., 2023; Schmidt and Felsche, 2024). These challenges are particularly pronounced in Bavaria, Germany’s largest federal state, where diverse agroecological gradients — ranging from alpine terrain to fertile lowlands — create heterogeneous growing environments that complicate biomass prediction (Bönecke et al., 2020; Dhillon et al., 2023a; Maestrini and Basso, 2018).

WW and OSR are economically and agronomically important crops in Bavaria (Statistik, 2020). Mapping their biomass patterns accurately is crucial for improving yield forecasting and informing landscape-level management decisions under climate uncertainty. Traditional light-use efficiency (LUE) models provide a physiologically grounded approach to estimating biomass from solar radiation and stress modifiers (e.g., temperature, vapor pressure deficit) (Dhillon et al., 2020, 2023c). However, they often fall short of capturing fine-scale spatial heterogeneity and the nonlinear effects of terrain, soil potential, and landscape structure on productivity (Riedesel et al., 2023; Stocker et al., 2018; Turner et al., 2002).

Recent advances in remote sensing (RS) and machine learning offer new pathways for overcoming these limitations. Random Forest (RF) models provide a flexible, non-parametric framework capable of incorporating multi-scale environmental variables, modeling complex interactions, and improving prediction accuracy across diverse landscapes (Breiman, 2001; Liaw and Wiener, 2002). When integrated with RS-derived biomass estimates, RF models can enhance both the mechanistic realism and spatial resolution of crop productivity assessments (Dhillon et al., 2023b; Leng and Hall, 2020; Peichl et al., 2021). This approach is valuable in regions such as Bavaria, where terrain complexity and climatic gradients create diverse growing conditions, and it may also be applicable in other areas with similar environmental heterogeneity and data availability.

Additionally, the structure of agricultural landscapes is increasingly recognized for its role in supporting ecosystem services that can contribute to crop yield stability and productivity (Tscharntke et al., 2012). Landscape metrics — such as the Shannon Diversity Index (SHDI), calculated to capture the diversity of land cover types, and the distribution of small woody features (SWFs) — influence microclimatic buffering, pollinator availability, and pest suppression (Dainese et al., 2019; Tamburini et al., 2020). These features capture the landscape complexity and have been associated with improved resilience in both temperate and tropical systems (Edwards et al., 2015; Grass et al., 2019). However, the extent to which these features modulate biomass variation in WW and OSR remains underexplored in Central European systems, particularly under varying climate conditions and from a geostatistical perspective.

Moreover, topographic variables, such as elevation, slope angle and aspect, alongside the soil potential (e.g., fertility, water-holding capacity) strongly influence biomass productivity by modulating local microclimates, water availability, and mechanization feasibility (Gessler et al., 1995; Maestrini and Basso, 2018). These environmental gradients interact with climate variability, shaping crop responses in spatially heterogeneous ways that are not clearly captured by traditional yield models (Bönecke et al., 2020).

Against this backdrop, this study aims to evaluate the predictive accuracy of a coupled approach that integrates RF with LUE-modeled biomass, comparing it against stand-alone LUE biomass estimates from Dhillon et al. (2023a) at the hexagon level and statistical yield data at the district level for the entire Bavaria. Additionally, we assess how biomass varies for WW and OSR in response to interacting environmental drivers across Bavaria, and determine the extent to which RF models explain biomass distribution across diverse landscapes and climatic conditions.

To achieve these aims, we integrate RS-derived biomass data with a suite of spatial and spatio-temporal predictors, including the Shannon Diversity Index (SHDI), small woody features (SWFs), topography, soil potential, and climate variability from 2001 to 2019, using a 5 km² hexagon-based spatial framework. To further interpret the influence of predictors on biomass outcomes across crops and regions, we apply SHapley Additive exPlanations (SHAP) values and Partial Dependence Plots (PDPs). This multi-dimensional approach offers insights into crop-specific responses to environmental heterogeneity and supports evidence-based agricultural planning under changing environmental conditions.

## 2 Materials and methods

### 2.1 Study area

**Figure 1:**
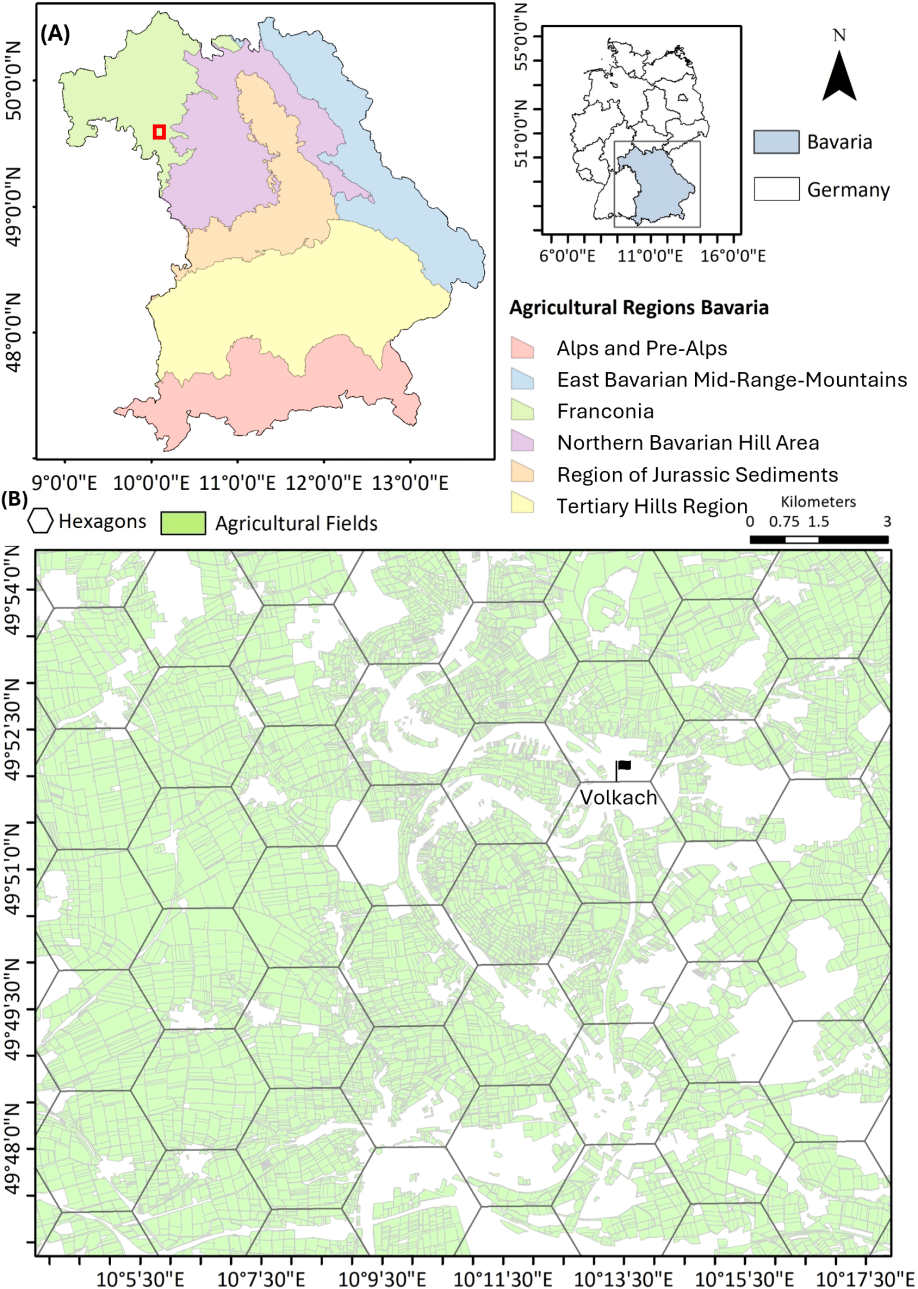
**(A)** Overview of the study region where the analysis is performed individually for Bavaria and its six agricultural areas: Alps and Pre-Alps, East-Bavarian Mid-Range Mountains, Franconia, Northern Bavarian Hill Area, Region of Jurassic Sediments, and Tertiary Hills Region. **(B)** The analysis is performed at hexagon levels on Bavaria’s land cover map (https://www.landklif.biozentrum.uni-wuerzburg.de/, accessed on 21 June 2021). Each hexagon has an area of 5 km^2^. Every hexagon is used to retrieve the field-based information on winter wheat and oilseed rape fields. The enlargement (displayed with a dark red box on the top-left map) shows the agricultural area of the village Volkach in Lower Franconia (northern Bavaria), with a peach-green color of agricultural fields in 2019.

This study focuses on Bavaria, the largest federal state in Germany, extending from 47°N to 50.5°N latitude and 9°E to 14°E longitude (**Figure 1**). Spanning approximately 70,550 km², about one-fifth of Germany’s land area, Bavaria features a highly heterogeneous terrain, ranging from the Bavarian Alps in the south to the Bavarian Forest and Fichtel Mountains in the east. It includes lowland regions such as the Franconian Basin in the north and the Danube River Valley traversing the central-southern part of the state. This spatial gradient significantly influences the regional climate, with mean annual temperatures ranging from -3.3°C to 11°C and annual precipitation sums from 500 mm in northern lowland areas to over 3,100 mm in the southern Alp (DWD, 1991–2020).

Forests cover 36.9% of Bavaria, while agricultural land accounts for 31.7%, primarily under cereal cultivation (Dhillon et al., 2022). According to LfStat (2020), WW occupied 23.9% of arable land, followed by silage and corn maize (21.4%), winter barley (11.4%), and summer barley (1.8%). OSR, grouped under “other field crops,” constitutes around 1%.

The analysis was structured around six distinct agricultural regions in Bavaria accounting for their agroecological and geomorphological conditions: the Alps and Pre-Alps, East-Bavarian Mid-Range Mountains, Franconia, Northern Bavarian Hill Area, Region of Jurassic Sediments, and Tertiary Hills Region (Wiesmeier et al., 2013; Wittmann, 1983; Würfl et al., 1984). Crop distribution varies across these regions. For example, the share of Bavaria’s total WW and OSR cultivation located in the Alps and Pre-Alps was minimal — 0.8% and 0.1%, respectively — while in the East-Bavarian Mid-Range Mountains, these shares were 2.0% and 0.7%. This limited presence is likely due to the cooler, wetter climate and rugged terrain in these areas. In contrast, with its warm, dry climate and fertile soils, Franconia supports 10.4% of WW and 1.8% of OSR. The Northern Bavarian Hill Area supported moderate cultivation (WW: 5.5%, OSR: 1.1%), while the Region of Jurassic Sediments (WW: 7.8%, OSR: 2.2%) and Tertiary Hills Region (WW: >20%) represented key regions with a large share of WW and OSR cultivation, supported by favorable soil and climatic conditions. These proportions represented the average field area between 2005 and 2019 [calculated from the field data by the Integrated Administration and Control System (IACS)].

### 2.2 Data

#### 2.2.1 Satellite-based biomass data

Biomass was estimated using an LUE model based on a fused Landsat–MOD13Q1 NDVI time series at 30 m resolution. The fusion employed the Spatial and Temporal Adaptive Reflectance Fusion Model (STARFM) algorithm (Gao et al., 2006) to produce an 8-day, cloud-free NDVI dataset (Dhillon et al., 2022). The LUE model incorporated solar radiation and stress scalars for minimum temperature (Tmin’), and vapor pressure deficit (VPD’) (Dhillon et al., 2023a) (**Equation 1**).


(1) $$\text{Biomass}=\;\sum_{SOS}^{EOS}\text{APAR}*\;({\text{Tmin}}^{'}{\text{*VPD}}^{'})*\;\in_{o}$$

In this equation, APAR is absorbed photosynthetically active radiation (MJ m^−2^ day^−1^), SOS and EOS are the start-and end of seasons of WW and OSR, and є_o_ is the optimal light-use efficiency (grams of carbon per megajoule of APAR (gC MJ^−1^)). The total aboveground biomass calculated by the LUE model is equivalent to the net primary productivity (NPP) (decitonnes (dt) ha−1 yr−1, with 1 dt ha^−1^ = 100 kg ha^−1^). Biomass was calculated per pixel for each crop-specific growing season: 15 February to 20 April for OSR (Zamani-Noor and Feistkorn, 2022) and 15 April to 30 June for WW (Harfenmeister et al., 2021), for each year from 2001 to 2019. Field boundaries and crop type information from the IACS database were used to identify WW and OSR pixels prior to LUE modeling, ensuring that biomass was estimated only within confirmed crop fields. All biomass values were expressed in dt ha^−1^, representing area-standardized estimates of productivity.

#### 2.2.2 Climate data

Daily climate variables, i.e., mean temperature (°C), precipitation (mm), and solar radiation (W m^−2^), were obtained from the Department of Applied Computer Science, Institute of Geography, University of Augsburg (https://www.uni-augsburg.de/de/fakultaet/fai/geo/prof/georkl/, accessed on 21 June 2021). These data were dynamically downscaled to 2,000 m resolution from ERA5 reanalysis using the hydrologically enhanced WRF model (Gochis et al., 2018; Hersbach et al., 2020; Skamarock et al., 2019). The dataset covered the 2001–2019 period and was aggregated by crop-specific growing seasons to calculate growing-season means and standard deviations (SDs) per year hexagon for each climate variable, as described in Section 2.3.1.

#### 2.2.3 Topography and soil potential

Topographic variables, including elevation (m a.s.l.), slope angle (degrees), and aspect (radians), were derived from the Shuttle Radar Topography Mission (SRTM) dataset at 30 m spatial resolution (Farr et al., 2007). Elevation data were accessed and processed in Google Earth Engine, where slope and aspect were calculated using the platform’s built-in terrain analysis functions.

Soil potential was derived from the Müncheberg soil quality ranking (SQR) dataset (Mueller et al., 2013), developed by the Leibniz Centre for Agricultural Landscape Research (ZALF) (https://www.bgr.bund.de/DE/Themen/Boden/Ressourcenbewertung/Ertragspotential/Ertragspotential_node.html, accessed on 10 December 2023). The SQR evaluates the long-term agricultural soil quality on a 0–100 scale, thus providing an approximation of the crop yield potential. It was available at 250 m resolution.

#### 2.2.4 IACS crop boundaries

Field-specific boundaries for WW and OSR were extracted from IACS data provided by the Bavarian State Institute for Agriculture (Bayerische Landesanstalt für Landwirtschaft, LfL) under a data agreement with the University of Würzburg. These shapefiles, available from 2005 to 2019, were used both to identify crop-specific pixels prior to LUE biomass modeling and to mask topographic, soil, and climate variables. This ensured that all analyses were strictly limited to areas cultivated with winter wheat or oilseed rape. For the years 2001 to 2004, the field boundaries of WW and OSR were reconstructed with the following approach: Pixels were identified for which the NDVI time series during the respective growing seasons closely matched the phenological profiles of these crops observed in 2005. NDVI data from fields delineated by the earliest available IACS boundaries were used for this. This step ensured consistency across the full 2001–2019 period and allowed us to capture long-term trends and interannual climate variability in biomass predictions.

#### 2.2.5 Land use/land cover data

The land use/cover (LULC) map used in this study was generated as part of the interdisciplinary Landklif project (https://www.landklif.biozentrum.uni-wuerzburg.de/, accessed on 21 June 2021) by integrating multiple datasets, including the Amtliche Topographisch-Kartographische Informationssystem (ATKIS), IACS, and Corine Land Cover (CLC, 100 m resolution). The LULC map represents land use conditions for 2019 and was developed for research purposes rather than as an official dataset. The classification process reclassified features from ATKIS, IACS, and CLC into six major land use categories: agriculture (annual crops, perennial crops, and managed grassland), forest (deciduous, coniferous, and mixed forest), grassland (managed and permanent grasslands), urban areas (settlements and traffic infrastructure), water bodies, and natural/semi-natural areas (hedgerows, wetlands, unmanaged grasslands, and succession areas). A hierarchical selection approach was applied to resolve spatial and thematic inconsistencies among IACS, ATKIS, and CLC datasets. IACS was prioritized for agricultural areas due to its high spatial accuracy and crop-specific detail; ATKIS for forest, grassland, urban, and water classes due to its detailed topographic data; and CLC to supplement natural and semi-natural areas. Where classification conflicts (e.g., the same parcel labeled differently across sources) or data gaps occurred, CLC was used to fill in missing or inconsistent information. The resulting harmonized land use/cover map formed the basis for calculating landscape metrics.

#### 2.2.6 Small woody features

Small woody features (SWFs) were derived from the Copernicus SWF dataset (2018) (https://land.copernicus.eu/pan-european/high-resolution-layers/small-woody-features/small-woody-features-2018, accessed 17 August 2023). The percentage of woody linear and patchy vegetation (e.g., hedgerows, tree rows) per hexagon was used to quantify local landscape complexity at 5 m resolution.

#### 2.2.7 *In-situ* crop yield data

District-level crop yield data for WW and OSR (dt ha^−1^) from 2001–2019 were obtained from the LfStat and used to validate modeled biomass outputs (https://www.statistikdaten.bayern.de/, which may require access credentials or institutional affiliation for data download, accessed on 21 June 2021). To validate the modeled biomass, hexagon-based RF-predicted biomass values were converted to yield estimates using crop-specific harvest indices: 0.48 for WW (Hay, 1995) and 0.30 for OSR (Diepenbrock, 2000). These yield estimates were then aggregated to the district level and compared to LfStat yield statistics using quadratic regression. Model performance was evaluated using the coefficient of determination (R²), root mean square error (RMSE), and normalized RMSE (NRMSE).

### 2.3 Methods

#### 2.3.1 Data preparation

**Figure 2:**
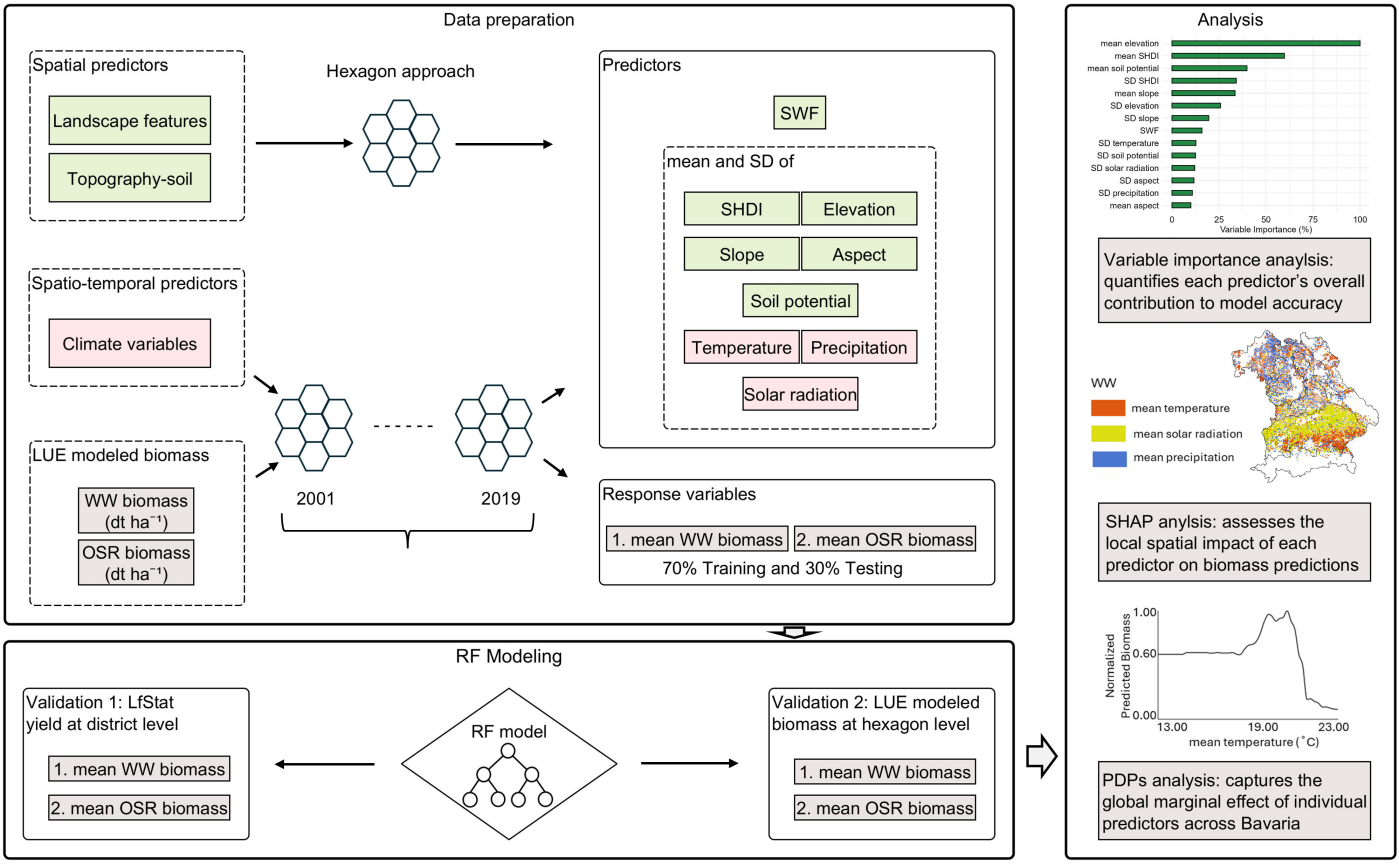
Overview of the data processing and modeling workflow for biomass prediction of winter wheat (WW) and oilseed rape (OSR) in Bavaria using Random Forest (RF) models. The upper panel illustrates the hexagon-based data preparation approach, integrating spatial predictors (landscape metrics, topography-soil) and spatio-temporal climate variables (temperature, precipitation, solar radiation) alongside LUE-modeled biomass for the period 2001 – 2019. Predictor variables include the mean and standard deviation (SD) of Shannon Diversity Index (SHDI), small woody features (SWF), topographic, soil, and climate factors. Biomass responses for WW and OSR were calculated per hexagon and used in RF modeling with a 70:30 train-test split. Model validation involved two steps: (1) comparing RF-modeled biomass to district-level yield statistics from LfStat, and (2) benchmarking RF against LUE-modeled biomass at the hexagon scale. The right panel presents the main analytical outputs: (i) variable importance analysis ranking predictor contributions to model accuracy, (ii) Bivariate SHapley Additive exPlanations (SHAP)-based spatial analysis revealing local predictor impacts across Bavaria, and (iii) Partial Dependence Plots (PDPs) showing the global marginal effects of key predictors (e.g., temperature) on biomass.

All datasets were spatially aligned to a hexagonal grid of 5 km² resolution covering Bavaria (**Figure 2**). The hexagon framework enabled spatial aggregation of predictors and response variables. Within each hexagon, biomass (dt ha^-1^) was first averaged annually per hectare and then across the 2001–2019 period, ensuring that values reflect mean biomass per unit area rather than total biomass for each hexagon and crop.

Climate variables (precipitation, temperature, and solar radiation) were first extracted as daily data from 2001 to 2019 and then aggregated for each crop’s growing season. For each year, growing-season precipitation sums, growing-season mean temperatures, and growing-season mean solar radiation were calculated, along with their respective SDs per hexagon. These annual growing-season metrics were then averaged across the 19 years to derive multi-annual mean and SD values per hexagon, serving as spatio-temporal predictors in the RF model.

Topographic variables (elevation, slope, aspect) and soil potential values represent static landscape features. The elevation and terrain data were originally developed in 2007, while soil potential maps were compiled in 2013. As these variables do not change annually, their values were assumed to be stable across the entire study period. Accordingly, they were extracted at their native spatial resolutions and aggregated to hexagon-level means and SDs, using crop-specific masks from 2005 to 2019 (based on IACS data) and from 2001 to 2004 (as reconstructed in Section 2.2.4). This allowed consistent spatial representation of topography and soil quality across the full 2001–2019 time series, enabling inclusion of early years that were essential for analyzing long-term climate variability and trends in biomass accumulation. The spatial distributions of climate predictors for WW and OSR are shown in **Figures A1** and **A2**, while the distributions of topographic and soil predictors are presented in **Figure A3**.

Landscape diversity was measured using the SHDI, which was computed on the 30 m resolution 2019 LULC map using a 9×9 moving window (270 m×270 m). SHDI values were then aggregated within each 5 km² hexagon based on five land cover classes: agriculture, forest, grassland, water, and natural/semi-natural areas. This window size was selected to balance local sensitivity with landscape-level smoothing, allowing the capture of relevant habitat heterogeneity without being overly influenced by small-scale noise. Compared to smaller windows (e.g., 3×3 or 7×7), the 9×9 window provided a more ecologically meaningful representation of land-cover diversity at the hexagon scale (Dhillon, 2023). SHDI values ranged from 0 (no diversity) to ~1.6, with values above 0.8 reflecting moderate to high land-cover diversity. The mean SHDI captured overall diversity, while the SD of SHDI reflected landscape heterogeneity and fragmentation (McGarigal et al., 2002; Wagner and Fortin, 2005). SWF (%) was calculated per hexagon from 5 m resolution data. The spatial distributions of mean SHDI, SD SHDI, and SWF (%) across Bavaria are presented in **Figure A4**.

#### 2.3.2 RF modeling

We selected the RF regression models due to its well-documented robustness in handling nonlinear relationships, multi-collinearity, and mixed data types without requiring strict assumptions about data distribution. RF has been widely used in agro-ecological studies for spatial prediction and variable importance analysis (Breiman, 2001; Dhillon et al., 2023b). Given our study’s focus on interpretability and the large number of spatial predictors, RF was a suitable and computationally efficient choice. Although alternative models such as gradient boosting or support vector machines exist, this study focused on RF as a representative and widely accepted machine learning approach.

RF regression models were applied separately for WW and OSR using the randomForest package in R (Liaw and Wiener, 2002). The response variable was multi-annual mean biomass, with predictors grouped as follows: (*i*) climate (mean and SD of temperature, solar radiation, and precipitation), (*ii*) topography and soil (mean and SD of elevation, slope angle and aspect, and soil potential), and (*iii*) landscape structure (mean and SD of SHDI and percent SWF cover). Model performance was evaluated using a 70:30 train-test split.

#### 2.3.3 Statistical analysis

Model performance was assessed through two validation steps: *(i)* RF-predicted biomass was converted to crop yield using harvest indices (0.48 for WW and 0.30 for OSR), averaged per district, and compared to official yield statistics from LfStat (as described in Section 2.2.7), and (*ii*) RF- and LUE-predicted biomass was compared at the hexagon scale. Model accuracy was quantified using: (*i*) the R² (**Equation 2**), (*ii*) the RMSE (**Equation 3**), and the NRMSE (**Equation 4**) calculated as:


(2) $${\text{R}}^{2}=1-\frac{\sum {\left({\text{O}}_{\text{i}}-{\text{P}}_{\text{i}}\right)}^{2}}{\sum {\left({\text{O}}_{\text{i}}-{\text{O}}^{\prime }\right)}^{2}}$$

(3) $$\text{RMSE}=\sqrt{\frac{1}{\text{n}}\sum_{\text{i}=1}^{\text{n}}{\left({\text{O}}_{\text{i}}-{\text{P}}_{\text{i}}\right)}^{2}}$$

(4) $$\text{NRMSE}=\frac{\text{RMSE}}{{\text{O}}^{\prime }}*100$$

In the equation, P_i_ is the predicted value, O_i_ is the observed value, O’ is the observed mean value, and n is the total number of observations. Variable importance was assessed using mean decrease in accuracy. To interpret the contribution of each predictor variable in the RF models, we applied SHAP and PDPs. SHAP values provide a local explanation by quantifying the influence of each predictor on individual predictions (i.e., per hexagon in our study). The values are derived from cooperative game theory and are normalized between 0 (no influence) and 1 (maximum influence), enabling spatial visualization of variable importance across Bavaria (Lundberg and Lee, 2017). This allows us to identify which variables matter most and where in the landscape they are most influential.

In contrast, PDPs offer a global explanation by showing the marginal effect of a predictor on the model’s output, averaged across the data. Each PDP curve illustrates how changes in a single predictor (e.g., mean solar radiation or slope angle) affect the predicted biomass when all other predictors are held constant (Friedman, 2001). This contributes to understanding the direction, threshold effects, and nonlinearity in the relationship between environmental variables and biomass. Together, these methods provide a comprehensive understanding of model behavior: SHAP values reveal where and how strongly variables matter locally, while PDPs show how variables relate to biomass production across their full value range.

## 3 Results

### 3.1 Model validation and key predictors of biomass

**Figure 3:**
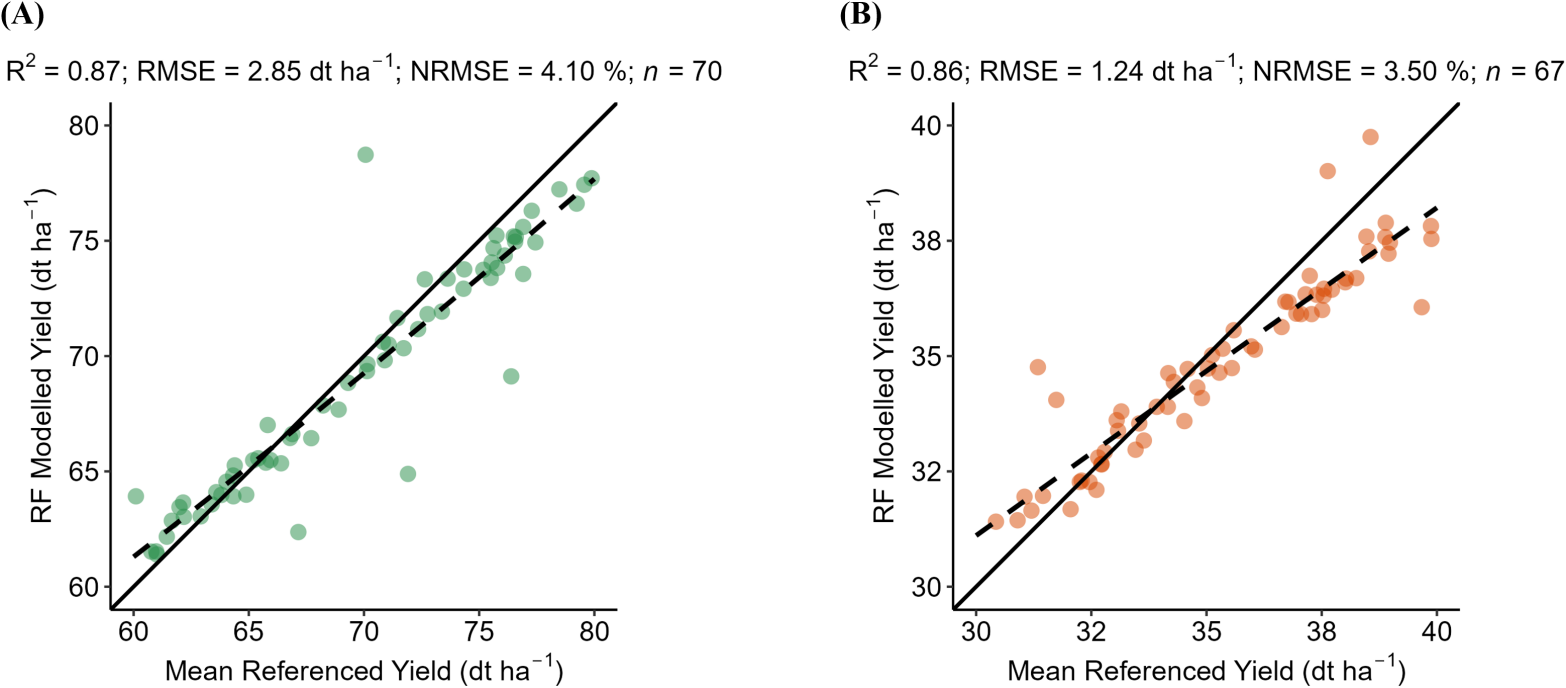
The scatter plots compare the RF-modeled yields (dt ha-1) against the mean of the district-level agricultural yields of **(A)** winter wheat (WW) and **(B)** oilseed rape (OSR) from the LfStat. The green dots represent WW. Orange dots represent OSR for each district. Plots contain a solid line to visualize the correlation of pixels between the referenced and modeled yield values. n represents the number of districts available to validate WW and OSR in Bavaria.

**Figure 4:**
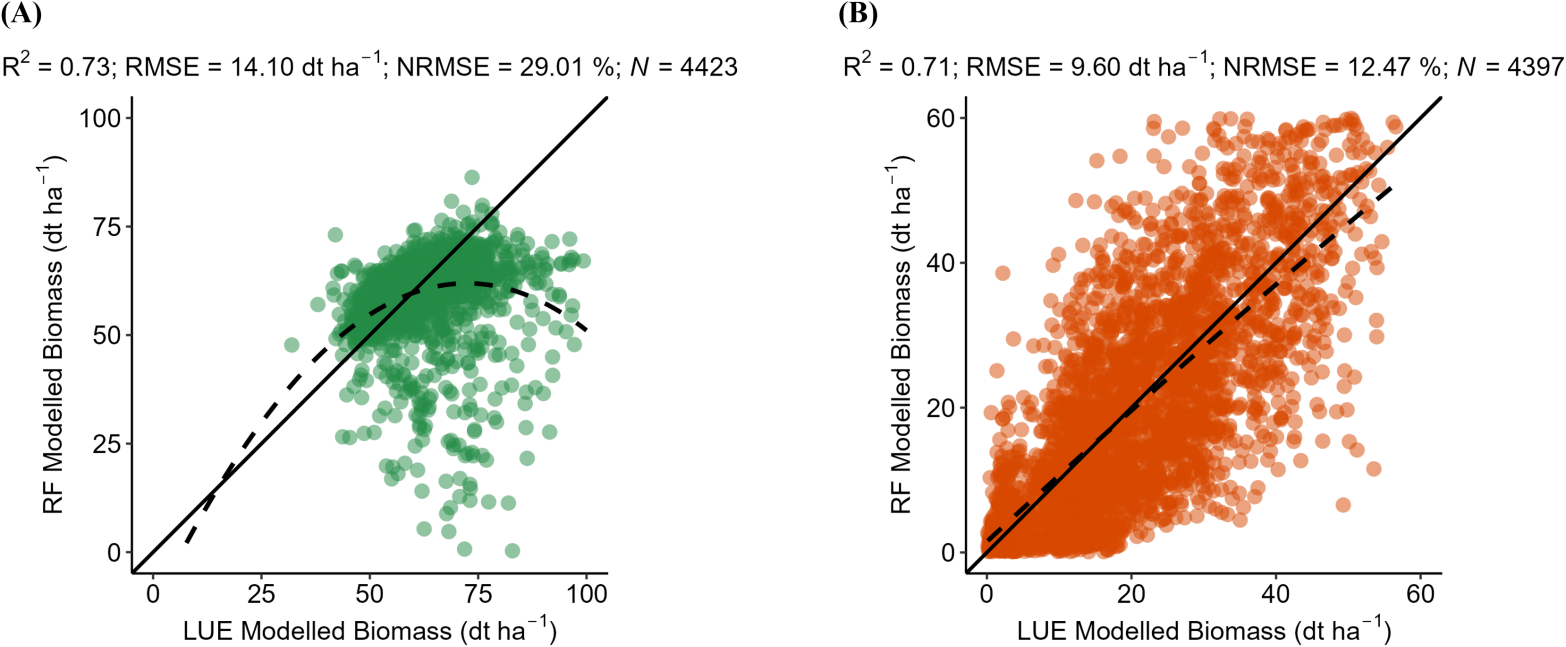
The scatter plots compare the RF-modeled biomass (dt ha^-1^) against the LUE-modeled biomass of **(A)** winter wheat (WW) and **(B)** oilseed rape (OSR). The green dots represent WW. Orange dots represent OSR for each hexagon. Plots contain a solid line to visualize the correlation of pixels between the referenced and modeled biomass values. N represents the number of hexagons available for validation for WW and OSR in Bavaria.

District-level yield validation quantifies the accuracy of the RF model. For WW, the model achieves an R^2^ of 0.87, RMSE of 2.85 dt ha^−1^, and NRMSE of 4.10% (**Figure 3A**). For OSR, the model yields an R^2^ of 0.86, RMSE of 1.24 dt ha^−1^, and NRMSE of 3.50% (**Figure 3B**). These results indicate a strong agreement between predicted and observed yields at the district level. This strong relationship likely stems from the integration of key agro-environmental predictors such as seasonal climate variability, landscape diversity, and soil potential, which collectively capture the main drivers of yield variation at the district level. The model is evaluated against mean LUE-derived biomass for 2001–2019 at the hexagon scale. The RF model attains an R² of 0.73 (RMSE = 14.10 dt ha^−1^) for WW and an R² of 0.71 (RMSE = 0.96 dt ha^−1^) for OSR (**Figures 4A, B**), reflecting substantial spatial agreement across both crops. However, the scatter plot for WW displays a compressed range, particularly at the lower biomass end. Only a few observations fall below 50 dt ha^−1^ and the model overestimates low biomass. In contrast, OSR shows a more continuous, near-normal distribution, suggesting more consistent agreement across the full biomass range. The model is evaluated against mean LUE-derived biomass from 2001 to 2019 at the hexagon level. For WW, the RF model achieved an R^2^ of 0.73, a RMSE of 14.10 dt ha^−1^, and a NRMSE of 29.01% (**Figure 4A**). For OSR, the model yielded an R^2^ of 0.71, RMSE of 9.60 dt ha^−1^, and NRMSE of 12.47% (**Figure 4B**). These results indicate substantial spatial agreement for both crops, with a lower relative prediction error for OSR. The scatter plot for WW shows a compressed range, particularly at the lower biomass end, where few observations fall below 50 dt ha^−1^ and the model tends to overestimate values. In contrast, OSR displays a more continuous distribution across the biomass range, indicating more consistent model performance and lower dispersion of errors.

**Figure 5:**
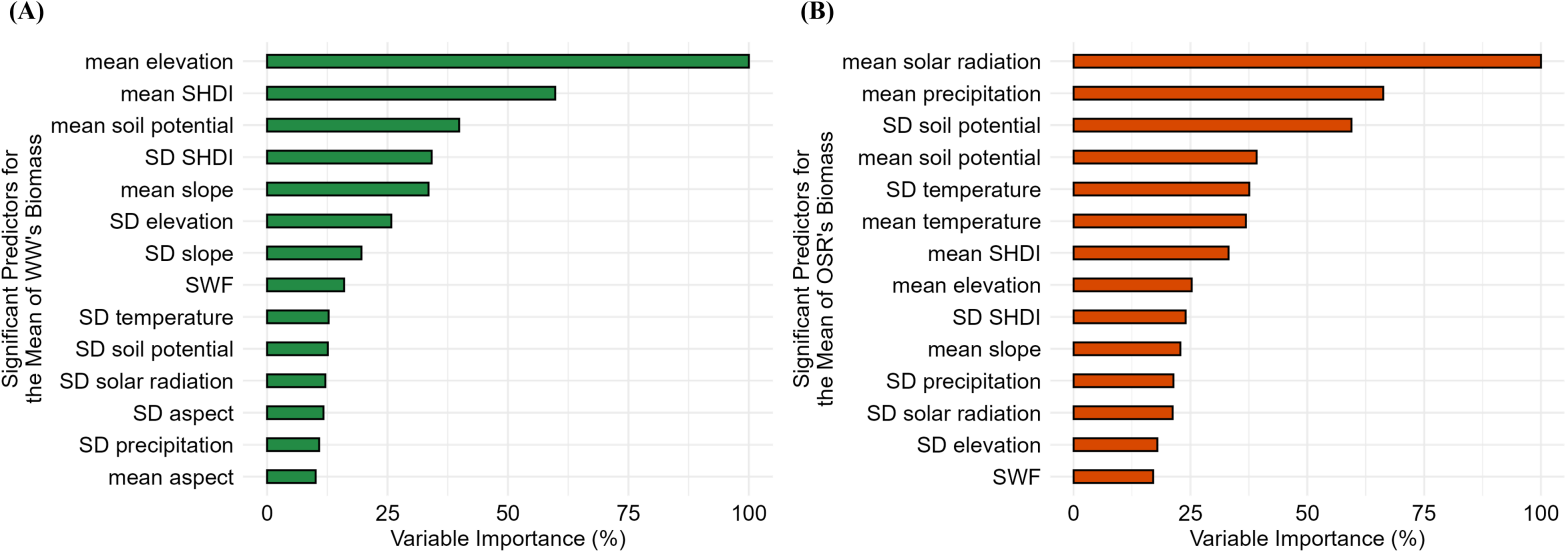
Variable importance for prediction of multi-annual mean biomass (2001-2019) at the hexagon level for winter wheat (WW) and oilseed rape (OSR). Panels **(A)** and **(B)** show the variable importance at the Bavarian scale for WW and OSR, respectively. Green bars represent WW, and orange bars represent OSR. SD refers to standard deviation, SWF refers to small woody features, and SHDI stands for the Shannon Diversity Index.

Variable importance analysis reveals crop-specific predictors of biomass. For WW, the mean elevation, SHDI, and soil potential are most influential (**Figure 5A**), indicating a strong influence of topography and landscape structure. For OSR, solar radiation, precipitation, and soil potential are the most important predictors (**Figure 5B**), suggesting greater sensitivity to climatic conditions.

### 3.2 Effects of landscape features on biomass distribution

**Figure 6:**
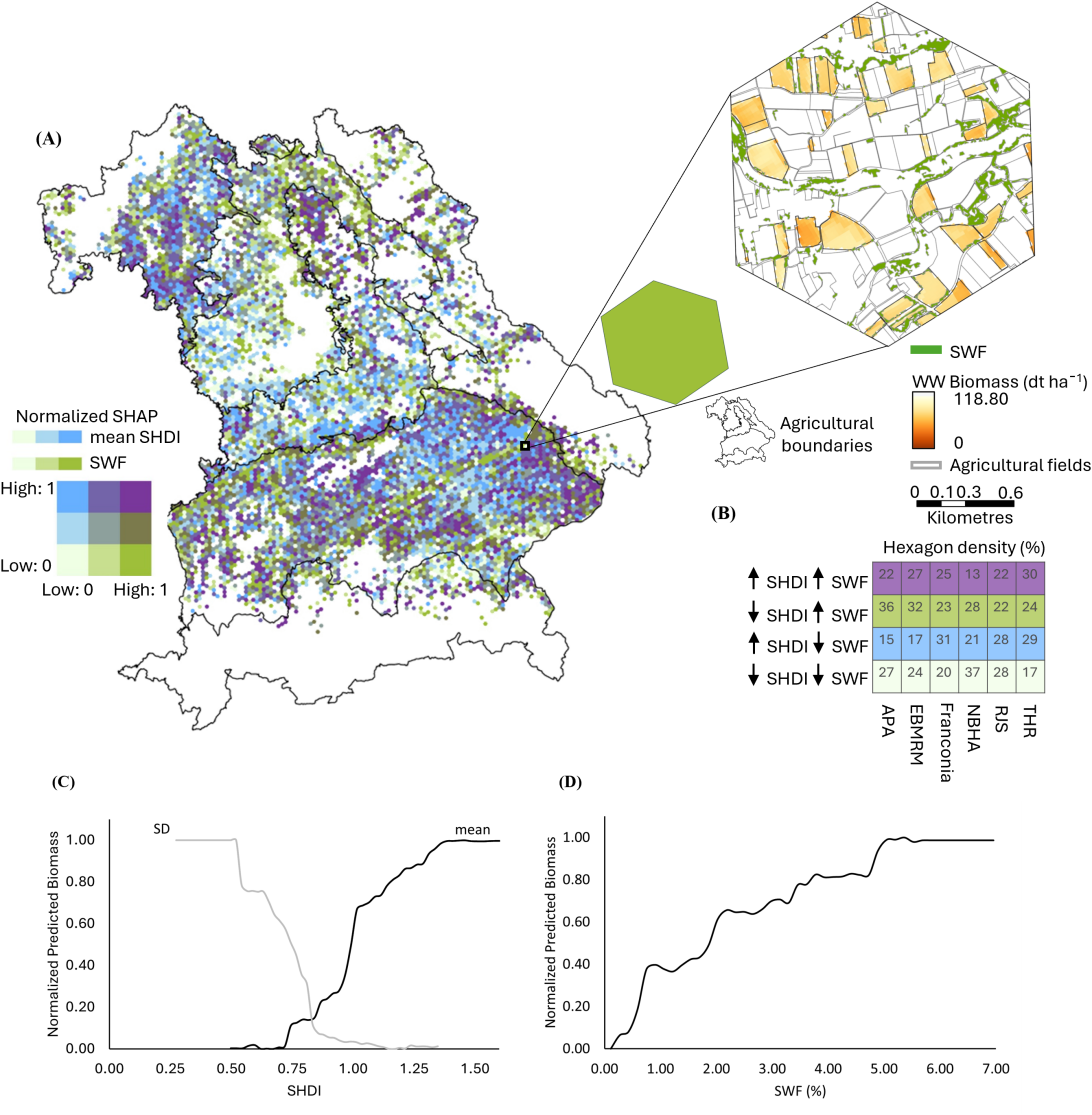
Spatial and statistical analysis of the influence of Shannon Diversity Index (SHDI) and Small Woody Features (SWF) on winter wheat (WW) biomass across Bavaria. **(A)** Bivariate SHapley Additive exPlanations (SHAP) map displaying normalized SHAP values (range: 0–1) for SHDI and SWF per hexagon, with higher values indicating stronger influence on WW biomass. A zoomed-in hexagon highlights an example where SWF has a high influence (green tone), while SHDI influence is low, suggesting a landscape with abundant woody elements but low overall land cover diversity. **(B)** Hexagon density matrix (%) showing the proportion of hexagons influenced by high/low SHDI and high/low SWF across Bavaria’s six agricultural regions: APA (Alps and Pre-Alps), EBMRM (East-Bavarian Mid-Range Mountains), Franconia, NBHA (Northern Bavarian Hill Area), RJS (Region of Jurassic Sediments), and THR (Tertiary Hills Region). **(C)** Partial Dependence Plots (PDPs) showing the effect of SHDI mean and standard deviation (SD) on predicted WW biomass. While mean SHDI indicates overall land cover diversity, the SD of SHDI reflects intra-hexagon variability, i.e., whether the landscape is uniformly diverse or contains spatial heterogeneity in patch types. **(D)** PDP showing the effect of SWF (%) on predicted biomass of WW. In both PDPs, predicted biomass values are normalized between 0 and 1, with 1 indicating maximum modeled biomass.

For WW, SHDI, expressed as a dimensionless index, has a strong influence in Franconia, the Region of Jurassic Sediments, and the Tertiary Hills Region (bluish colors in **Figure 6A**). Biomass increases where SHDI exceeds 0.8 and plateauing at 1.4 (**Figure 6C**). High variability (SD > 0.6) in SHDI is associated with reduced biomass. SHAP analysis determines the positive effect of moderate woody element presence (greenish colors in **Figure 6A**). SWFs have the most decisive influence in the East-Bavarian Mid-Range Mountains, the Northern Bavarian Hill Area, and the Alps and Pre-Alps. The PDP reveals that predicted biomass increases with SWF coverage, reaching a plateau at approximately 7% SWF cover, indicating a positive but saturating effect of woody elements on WW productivity (**Figure 6D**). Around 25% of hexagons in Franconia, 27% in the East-Bavarian Mid-Range Mountains, and 30% in the Tertiary Hills Region show co-influence from both landscape metrics (**Figure 6B**).

**Figure 7:**
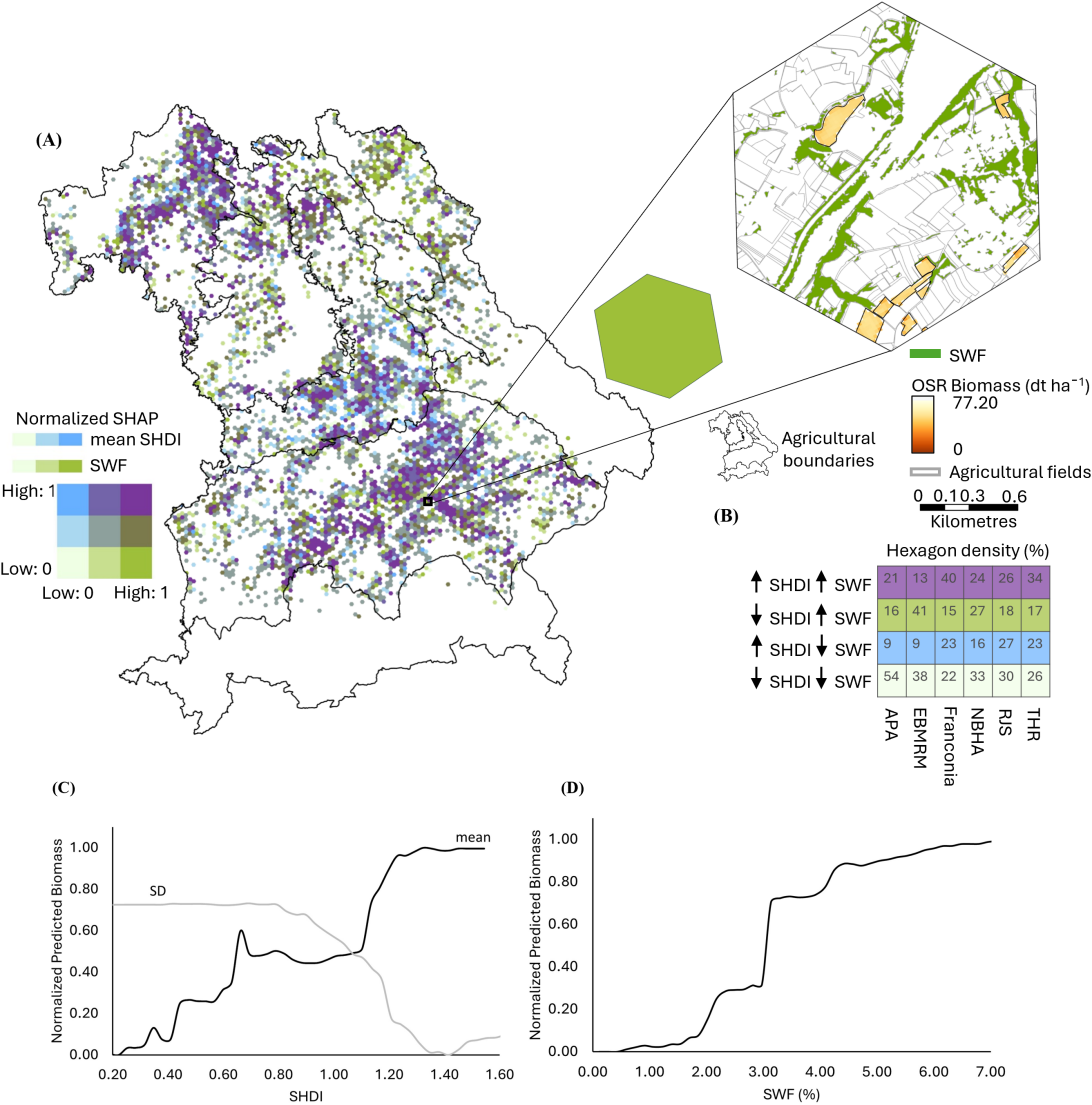
Spatial and statistical analysis of the influence of Shannon Diversity Index (SHDI) and Small Woody Features (SWF) on oilseed rape (OSR) biomass across Bavaria. **(A)** Bivariate SHapley Additive exPlanations (SHAP) map displaying normalized SHAP values (range: 0–1) for SHDI and SWF per hexagon, with higher values indicating stronger influence on WW biomass. A zoomed-in hexagon highlights an example where SWF has a high influence (green tone), while SHDI influence is low, suggesting a landscape with abundant woody elements but low overall land cover diversity. **(B)** Hexagon density matrix (%) showing the proportion of hexagons influenced by high/low SHDI and high/low SWF across Bavaria’s six agricultural regions: APA (Alps and Pre-Alps), EBMRM (East-Bavarian Mid-Range Mountains), Franconia, NBHA (Northern Bavarian Hill Area), RJS (Region of Jurassic Sediments), and THR (Tertiary Hills Region). **(C)** Partial Dependence Plots (PDPs) showing the effect of SHDI mean and standard deviation (SD) on predicted WW biomass. While mean SHDI indicates overall land cover diversity, the SD of SHDI reflects intra-hexagon variability, i.e., whether the landscape is uniformly diverse or contains spatial heterogeneity in patch types. **(D)** PDP showing the effect of SWF (%) on predicted biomass of WW. In both PDPs, predicted biomass values are normalized between 0 and 1, with 1 indicating maximum modeled biomass.

For OSR, SHDI has the greatest influence in Franconia and the Tertiary Hills Region (bluish colors in **Figure 7A**), while SWFs exert a stronger influence in the Alps, Pre-Alps and the East-Bavarian Mid-Range Mountains (greenish colors in **Figure 7A**). Around 40% of hexagons in Franconia, 34% in the Tertiary Hills Region, and 26% in the Region of Jurassic Sediments show co-influence from SHDI and SWF (**Figure 7B**). Biomass increases with SHDI up to approximately 1.4 but declines sharply when variability (SD) exceeds 0.8, indicating a negative effect of highly fragmented landscapes (**Figure 7C**). This negative effect at high SD values may partially reflect a statistical artifact, as hexagons with high mean SHDI often consist of uniformly diverse land-cover compositions, which inherently constrain further variability. However, such configurations also reduce the dominance of productive crop land, potentially lowering biomass, and the RF model captures this relationship through its hierarchical structure. The predicted biomass consistently rises with increasing SWF coverage, reaching a plateau near 7%, reflecting a positive but saturating effect of woody elements on OSR productivity (**Figure 7D**).

### 3.3 Climate influence on biomass distribution

**Figure 8:**
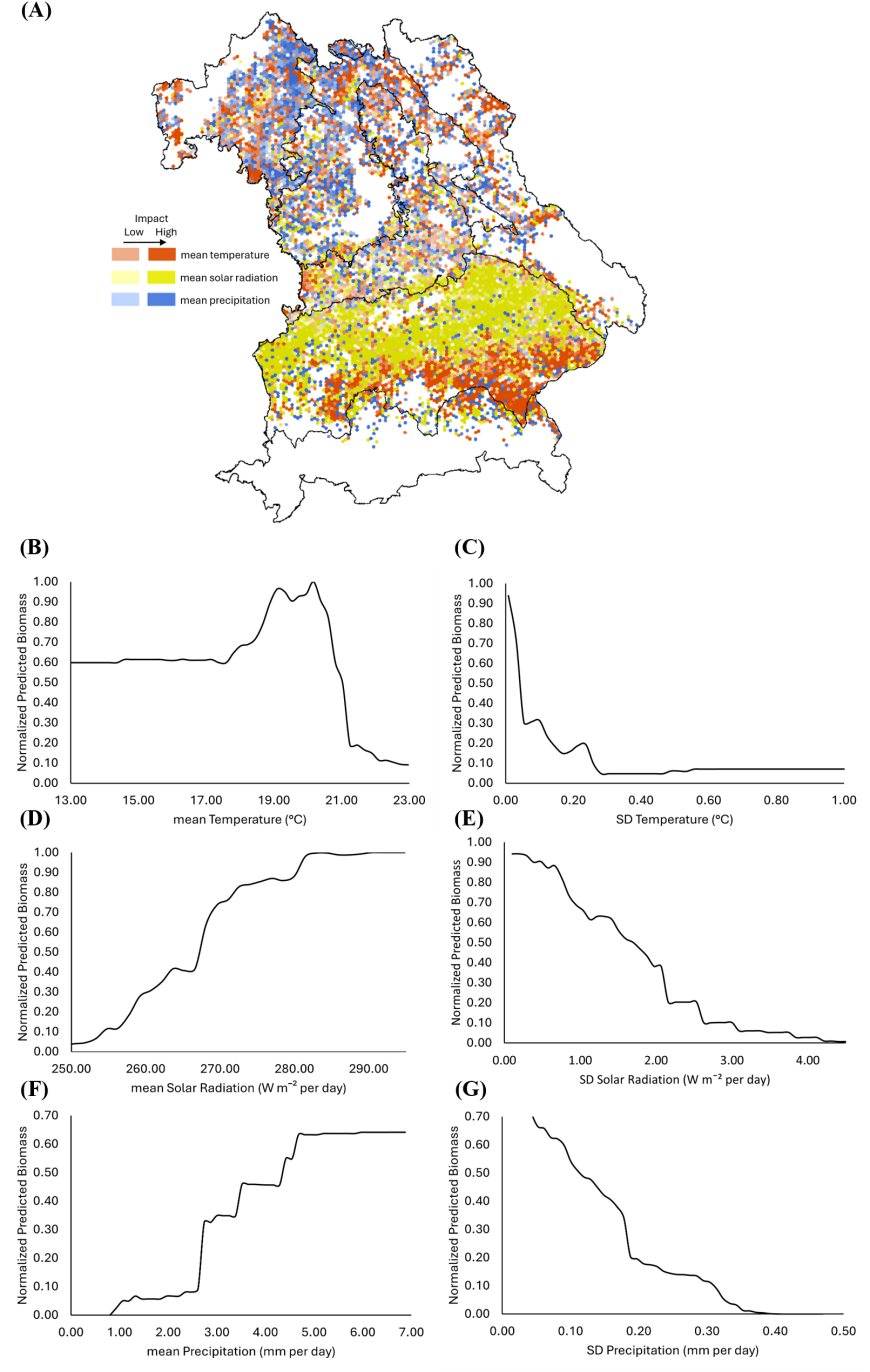
Spatial and functional impact of climate variables on winter wheat (WW) biomass across Bavaria. **(A)** SHapley Additive exPlanations (SHAP)-based spatial distribution of the influence of mean temperature (red), mean solar radiation (yellow), and mean precipitation (blue) on predicted WW biomass. **(B–G)** Partial Dependence Plots (PDPs) showing the relationship between normalized predicted biomass and **(B)** mean temperature, **(C)** standard deviation (SD) of temperature, **(D)** mean solar radiation, **(E)** SD of solar radiation, **(F)** mean precipitation, and **(G)** SD of precipitation. In all PDPs, predicted biomass values are normalized between 0 and 1, with 1 indicating maximum modeled biomass.

Solar radiation emerges as the most important climatic predictor for WW, especially in the Tertiary Hills Region (**Figure 8A**). Biomass increases with mean solar radiation, plateauing at ~280 W m^−2^ per day (**Figure 8D**), while variability above 40 W m^−2^ per day reduces biomass (**Figure 8E**). Temperature positively influences biomass between 17 – 20°C, with declines beyond 21°C (**Figure 8B**). Even slight interannual variability in temperature, exceeding just 0.1 – 0.2 °C, appears to substantially reduce predicted biomass, highlighting the crop’s sensitivity to temperature fluctuations (**Figure 8C**). Precipitation above 2 mm day^−1^ supports higher biomass (**Figure 8F**), but variability exceeding 0.2 mm day^−1^ has a negative impact (**Figure 8G**). These thresholds represent model-derived inflection points from PDP analysis under the 2001–2019 conditions in Bavaria.

**Figure 9:**
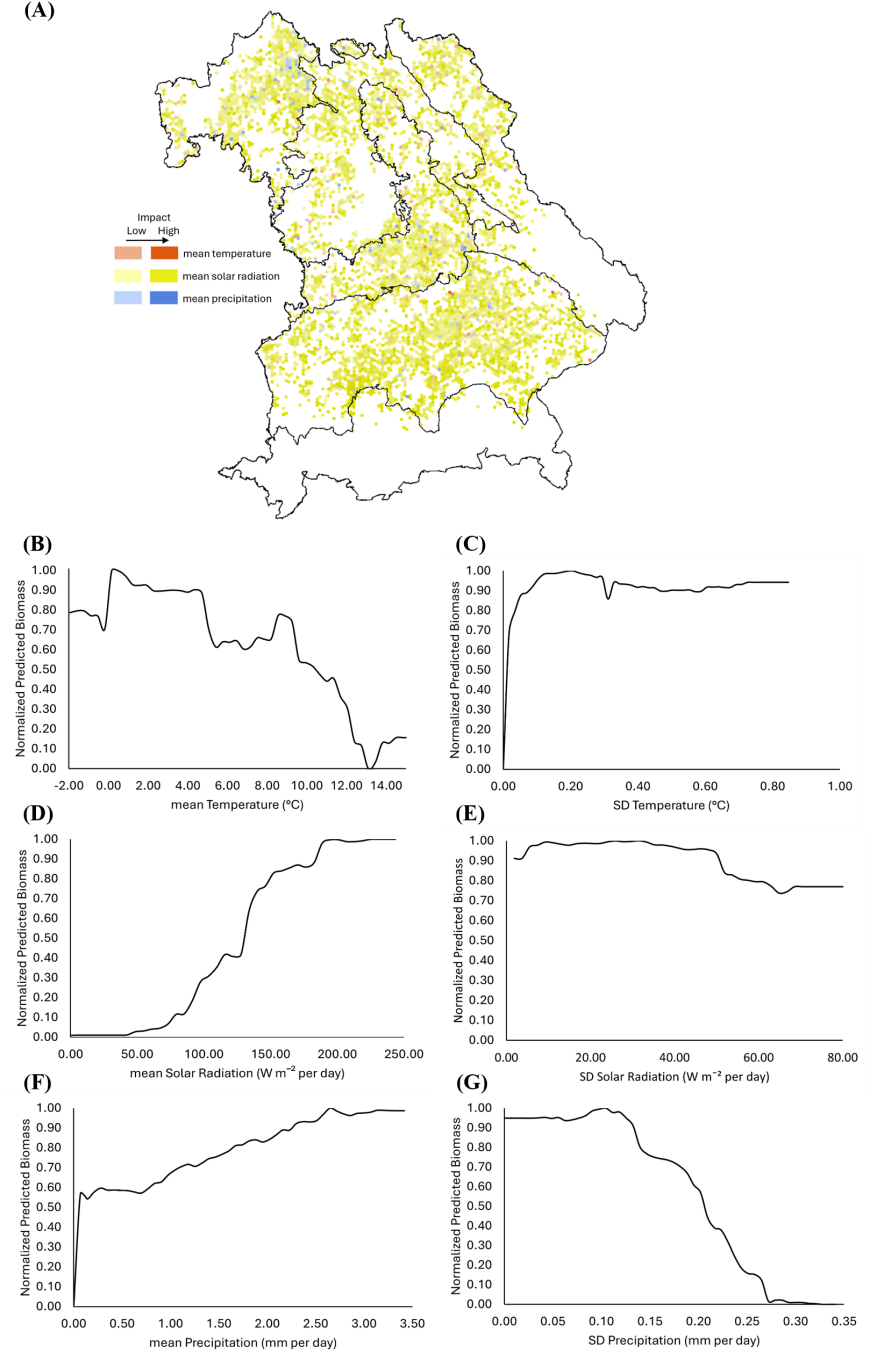
Spatial and functional impact of climate variables on oilseed rape (OSR) biomass across Bavaria. **(A)** SHapley Additive exPlanations (SHAP)-based spatial distribution of the influence of mean temperature (red), mean solar radiation (yellow), and mean precipitation (blue) on predicted WW biomass. **(B–G)** Partial Dependence Plots (PDPs) showing the relationship between normalized predicted biomass and **(B)** mean temperature, **(C)** standard deviation (SD) of temperature, **(D)** mean solar radiation, **(E)** SD of solar radiation, **(F)** mean precipitation, and **(G)** SD of precipitation. In all PDPs, predicted biomass values are normalized between 0 and 1, with 1 indicating maximum modeled biomass.

For OSR, solar radiation is the most influential climatic driver (**Figure 9A**). Biomass increases with solar radiation up to ~250 W m^−2^ per day (**Figure 9D**), with little gain beyond this value. Temperature stability is associated with higher biomass, but declines are evident when the mean temperature exceeds 12°C (**Figure 9B**). Precipitation has positive effects above 1.5 mm day^−1^ (**Figure 9F**), while high variability (SD > 0.15 mm day^−1^) corresponds to reduced biomass (**Figure 9G**). In contrast to WW, variability in temperature and solar radiation shows little effect on OSR biomass (**Figures 9C, E**). This may reflect OSR’s earlier growing season (mid-February to April), during which climatic conditions are typically more stable. As with WW, the thresholds represent model-based responses specific to the 2001–2019 climatic conditions in Bavaria and are not intended as universal physiological limits.

### 3.4 Role of soil and topography in biomass patterns

**Figure 10:**
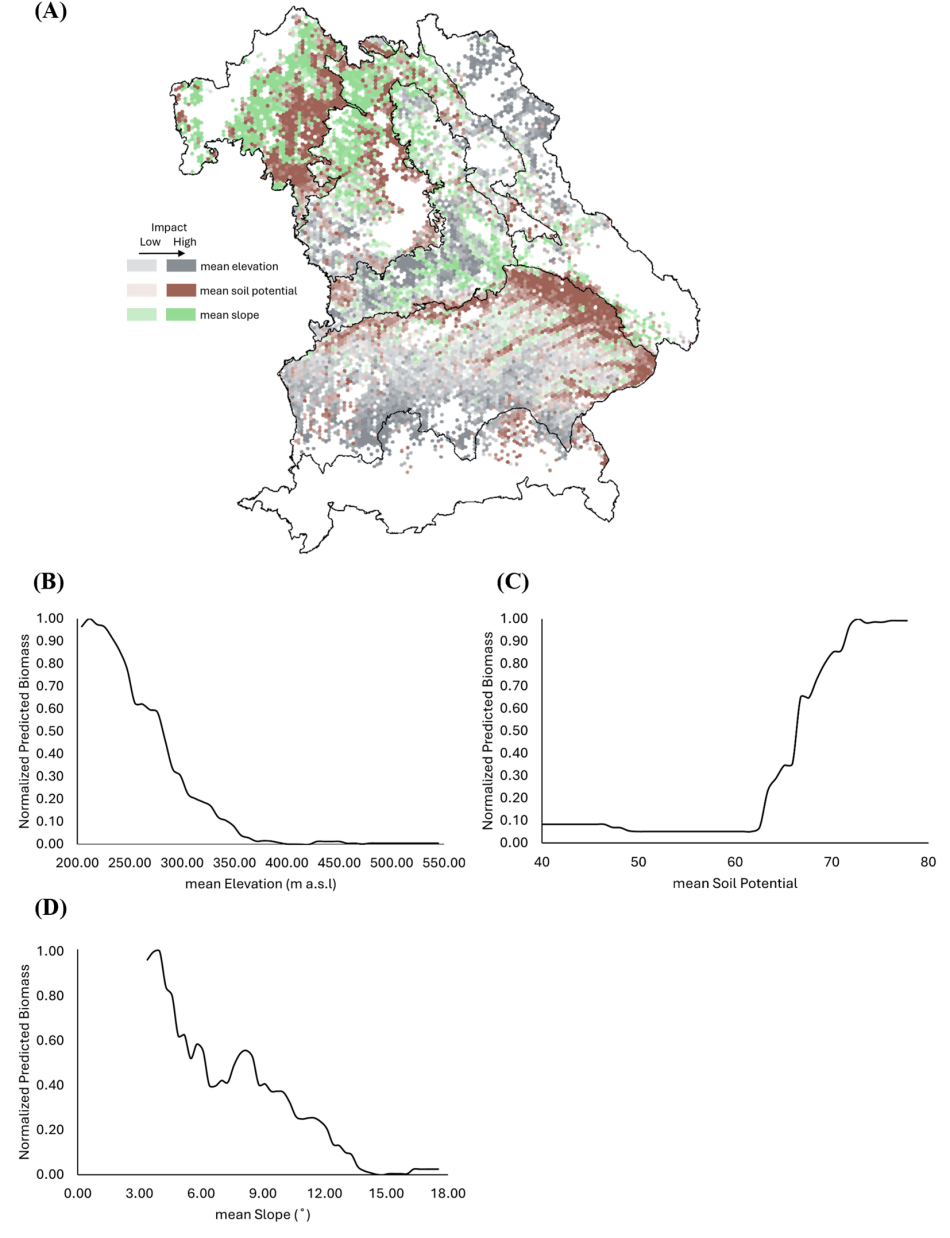
Spatial influence of soil-topography factors on the predicted biomass of winter wheat (WW) across Bavaria. **(A)** SHapley Additive exPlanations (SHAP)-based analysis illustrating the impact of mean elevation (gray), mean soil potential (brown), and mean slope (green) on biomass prediction. Higher intensity colors indicate a stronger influence of the respective factors. **(B–D)** Partial dependence plots (PDPs) showing the relationship between predicted biomass and **(B)** mean elevation (m a.s.l.), **(C)** mean soil potential, and **(D)** mean slope (°). In all PDPs, predicted biomass values are normalized between 0 and 1, with 1 indicating maximum modeled biomass.

Across Bavaria, the biomass of WW decreases with increasing elevation, particularly beyond 300 m a.s.l., and stabilizes at lower values beyond 400 m (**Figure 10B**). Slope angle shows an adverse effect on biomass, with reductions starting beyond 3°, and further declines beyond 8° (**Figure 10D**). In contrast, biomass positively responded to soil potential, increasing sharply above an SQR score of 60 and plateauing near 75 (**Figure 10C**). This reflects the soil’s capacity to support high productivity under favorable conditions. SHAP values indicate that soil potential exerts a particularly strong influence on biomass in Franconia and the Tertiary Hills Region. At the same time, elevation emerges as a key factor in areas with pronounced altitudinal gradients, including the Alps, East-Bavarian Mid-Range Mountains, and the Region of Jurassic Sediments, where its impact on biomass is notably higher (**Figure 10A**).

**Figure 11:**
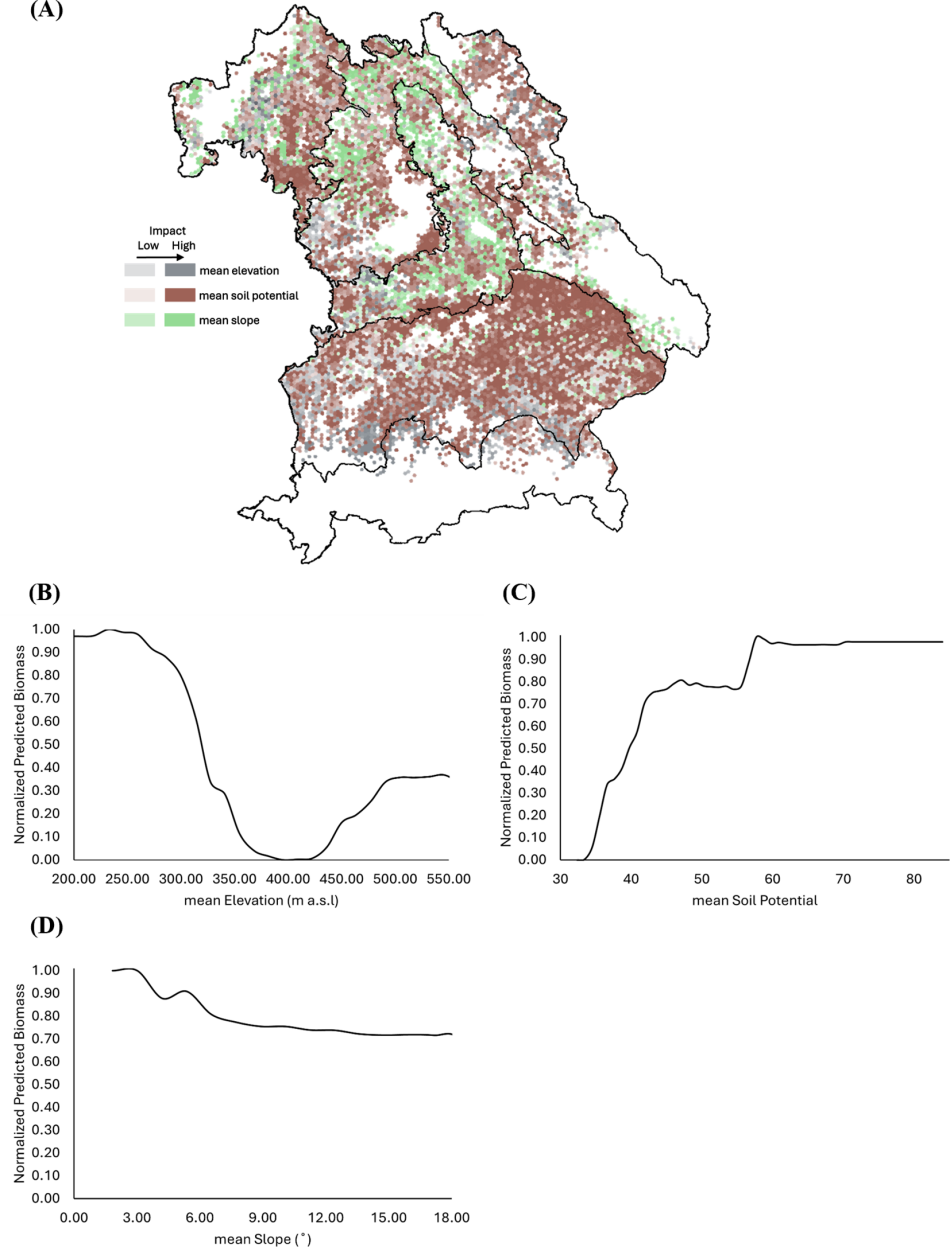
Spatial influence of soil-topography factors on the predicted biomass of oilseed rape (OSR) across Bavaria. **(A)** SHapley Additive exPlanations (SHAP)-based analysis illustrating the impact of mean elevation (gray), mean soil potential (brown), and mean slope (green) on biomass prediction. Higher intensity colors indicate a stronger influence of the respective factors. **(B–D)** Partial dependence plots (PDPs) showing the relationship between predicted biomass and **(B)** mean elevation (m a.s.l.), **(C)** mean soil potential, and **(D)** mean slope (°). In all PDPs, predicted biomass values are normalized between 0 and 1, with 1 indicating maximum modeled biomass.

OSR biomass shows a milder decline with elevation, with the lowest values occurring between 350–400 m above sea level, followed by a slight recovery above 450 m (**Figure 11B**). The effect of slope angle is weaker than for WW, with only a slight reduction in biomass occurring beyond 8° (**Figure 11D**). Biomass increased with soil potential above 50, reaching a peak near an SQR score of 75 (**Figure 11C**). SHAP analysis confirms that soil potential plays a central role across all oilseed rape-growing regions, while slope shows minimal spatial influence (**Figure 11A**).

## 4 Discussion

### 4.1 Advantages of coupling RF with LUE for biomass prediction

Our study demonstrates that coupling the RF regression model with LUE-derived biomass outputs improves predictive accuracy compared to using the LUE model alone. While LUE models are widely applied for estimating annual biomass based on physiological constraints such as solar radiation, temperature stress, and vapor pressure deficit, they are limited in capturing the environmental heterogeneity’s spatial and temporal variability (Turner et al., 2002). The coupled RF approach leverages mechanistic process understanding and data-driven pattern recognition by integrating LUE-modeled biomass as the response variable and enriching the model with a broader set of predictors. The predictors included the mean and SD of spatial variables (e.g., SHDI, elevation, slope, aspect, and soil potential) and spatio-temporal climate variables (mean and SD of temperature, solar radiation, and precipitation) from 2001 to 2019. Compared to the stand-alone LUE model (R² = 0.79, RMSE = 4.51 dt ha^−1^ for WW), the coupled RF-LUE approach improved accuracy by ~10% and reduced RMSE by 36% (Dhillon et al., 2023a). For OSR, while R² remained at 0.86, coupling nearly halved the RMSE, highlighting the added value of combining physiological modeling with machine learning. These findings are consistent with prior results from our earlier study, where a hybrid RF model using LUE-derived biomass achieved a ~14% improvement in R² and reduced error relative to the LUE model alone (Dhillon et al., 2023b). However, that study was limited to the year 2019, used only climate variables as predictors, and was conducted at the district level. In contrast, the current study spans a longer period (2001–2019), integrates additional landscape, topographic, and soil predictors, and applies a finer-scale hexagon-based spatial framework for improved resolution and generalizability. Similar outcomes were reported by Shahhosseini et al. (2021), who showed that integrating outputs from process-based crop models into machine learning frameworks reduced yield prediction errors by 7–20% across the U.S. Corn Belt. Overall, integrating RF with LUE offers a more robust modeling framework by capturing nonlinear responses and threshold effects and better accounting for spatial variation across landscapes (De’ath and Fabricius, 2000).

While the hybrid RF-LUE approach substantially improved prediction accuracy, further analysis of the validation scatter plots revealed crop-specific differences in model behavior. For OSR, the modeled biomass values followed a relatively continuous and near-normal distribution across the full biomass range, suggesting consistent predictive performance. In contrast, the WW scatter plot displayed a compressed distribution, with few low-biomass hexagons and an overestimation of low values. This discrepancy may arise from limitations in the LUE model’s ability to represent low biomass conditions for WW, potentially linked to NDVI saturation effects, phenological misclassification, or unaccounted environmental stressors. These observations emphasize that, in addition to global accuracy metrics such as R² and RMSE, the distributional characteristics of model predictions provide valuable insights into model performance and should be considered in future model refinements.

Although RF models require careful tuning to avoid overfitting, coupling them with mechanistic crop models improves both interpretability and accuracy (Segal, 2004; Shahhosseini et al., 2021). This hybrid approach holds promise for advancing large-scale, spatially explicit biomass estimation under diverse environmental conditions.

### 4.2 Landscape effects on biomass predictions

Our findings reinforce a growing body of evidence that landscape structures, specifically landscape diversity and the presence of SWFs, play a critical role in predicting crop biomass production across diverse agricultural systems. These results are consistent with landscape-scale studies demonstrating how land-use diversity enhances ecosystem resilience by buffering crops against environmental stressors (Nelson and Burchfield, 2021). For instance, analyses across counties in the USA reported that increased landscape complexity can boost corn and wheat yields by up to 20%, an effect attributed to pollination, pest regulation, and microclimatic moderation improvements (Nelson and Burchfield, 2021).

The role of SWFs in enhancing crop stability is also well-documented in agroecological literature. The stabilizing effect of SWFs on biomass observed in this study is likely due to their capacity to buffer microclimatic extremes and support beneficial arthropod populations. Previous research has shown that woody features, such as hedgerows and tree lines serve as habitats for pollinators and natural enemies of pests (Albrecht et al., 2020). They also reduce wind exposure and enhance soil moisture retention, factors especially critical during dry periods. Galpern et al. (2020) showed that even small additions of complex land cover at field boundaries can increase yield outcomes. Similarly, Nguyen et al. (2022) used RS to demonstrate that yields in canola fields were higher up to 100 meters from structurally complex field margins in a 100 × 100 km area of Alberta, Canada—a finding that resonates with our results. In our study, higher biomass levels of WW and OSR are predicted in regions of Bavaria (approximately 70,550 km²) characterized by greater landscape diversity and increased presence of SWFs, indicating that such structural complexity supports higher biomass at a much broader spatial scale.

Importantly, our results underscore the crop-specific nature of landscape effects. While both WW and OSR benefitted from moderate to high SHDI and SWF coverage, OSR appeared more sensitive to variation in landscape structure—particularly as represented by the spatial distribution and diversity of land cover types. This heightened sensitivity does not necessarily reflect a response to environmental fluctuations (e.g., climatic variability), but rather suggests that oilseed crops may be more influenced by local habitat configuration, potentially due to their narrower ecological tolerances and more complex phenological requirements (Ahmad et al., 2021). In contrast, WW demonstrates less sensitivity to spatial variation in landscape structure, which may reflect the broader ecological adaptability often observed in cereal crops (Baldoni et al., 2021). Regionally, this was most apparent in Franconia and the Tertiary Hills Region, where higher SHDI and moderate SWF coverage coincided with greater biomass production, while the more fragmented Alpine and East-Bavarian Mid-Range Mountains regions showed lower performance under similar conditions.

Interestingly, we also observed a plateau in the relationship between biomass and the highest values of SHDI or SWF. This suggests that beyond a certain threshold, increasing structural complexity does not necessarily lead to further biomass gains. One possible explanation lies in management constraints associated with highly structured agricultural landscapes: smaller fields, irregular shapes, and dense boundary features can limit the efficiency of mechanized farming, reduce field-level management intensity, or complicate crop rotation strategies. These landscapes may also reflect less intensive farming systems (e.g., smallholder or low-input agriculture), which could naturally yield lower biomass despite their ecological benefits. Thus, while moderate levels of landscape complexity appear beneficial, very high values may not always correspond to the highest productivity levels.

Collectively, these results highlight the value of incorporating spatial landscape metrics into biomass modeling frameworks. When used alongside climatic and edaphic predictors, metrics such as SHDI and SWF can enhance our ability to predict and manage spatial variability in crop performance. Furthermore, they lend empirical support to the principles of ecological intensification: maintaining structurally diverse agricultural landscapes not only supports biodiversity but also improves yield stability under increasing climatic variability.

### 4.3 Climate variability as an important driver of biomass prediction

Our study quantifies that both mean climatic conditions and their interannual variability are critical drivers of crop biomass distribution across Bavaria. While solar radiation, temperature, and precipitation set the baseline for photosynthetic activity and biomass accumulation, their year-to-year fluctuations play an outsized role in destabilizing yields. This trend is particularly evident in regions with pronounced seasonal shifts. In this regard, regions like the Tertiary Hills Region and Franconia, with favorable but seasonally variable climatic regimes, showed higher biomass sensitivity to fluctuations in solar radiation and precipitation. A likely reason is that their relatively high productive potential is more easily disrupted by inconsistent weather.

The pronounced sensitivity of WW biomass to fluctuations in solar radiation, temperature, and precipitation reflects its physiological dependence on consistent energy and water availability. In contrast, OSR biomass appears less sensitive to interannual variability in solar radiation and temperature, which may be explained by differences in phenology. Compared to WW, the limited sensitivity of OSR biomass to these variables likely stems from its earlier growing season, from mid-February to April, which coincides with a climatologically more stable period. This temporal mismatch with the more variable spring and summer conditions may reduce OSR’s exposure to weather extremes, weakening the influence of climatic variability in the RF model. However, OSR responds notably to precipitation variability, highlighting crop-specific climatic sensitivities. These results align with global-scale studies that estimate approximately one-third of yield variability is attributable to climate variability, especially in temperature and precipitation (Ray et al., 2015; Vogel et al., 2019). For instance, heatwaves and droughts during key phenophases have been shown to account for a majority of yield losses in cereals like wheat and maize (Lesk et al., 2016), while erratic rainfall patterns can lead to synchronous crop failures across broad geographic areas (Mehrabi and Ramankutty, 2019).

In our study, solar radiation emerges as the most influential climatic driver for OSR based on SHAP analysis, as indicated by the dominant blue shading across Bavaria in **Figure 9A**. This spatially explicit pattern shows that solar radiation has a stronger and more widespread influence than other climate variables. These findings corroborate previous research emphasizing radiation stability as a core determinant of yield resilience, particularly in photosynthetically intensive crops (Tack et al., 2015). Likewise, temperature effects were non-linear. WW and OSR exhibited crop-specific thresholds beyond which biomass declined, suggesting different thermal tolerances and reproductive sensitivities. This observation is consistent with modeling and field-based studies that link yield instability to extreme temperature events and poor thermal adaptation (Zhao et al., 2017).

In Germany, the differentiated responses of WW to precipitation variability also echo earlier studies that highlight how moisture inconsistency, not just total rainfall, is a major determinant of yield outcomes (Becker et al., 2025). While OSR appeared slightly more tolerant to fluctuations, both crops exhibited substantial declines under high variability, emphasizing the importance of rainfall consistency for biomass accumulation.

Overall, the findings underscore that climatic variability, not just mean conditions, must be explicitly incorporated into biomass prediction models. Metrics such as the SD of solar radiation, temperature, and precipitation offer crucial insights into crop sensitivity and potential adaptation pathways. As climate extremes become more frequent under global change (e.g., in Franconia (Paeth et al., 2023)), integrating such variability metrics can improve model robustness and support the design of climate-smart agricultural systems. Future resilience strategies should consider both physiological thresholds and crop-specific climate sensitivities alongside interventions such as drought-tolerant cultivars, adjusted sowing dates, and supplemental irrigation to buffer against the impacts of erratic weather patterns.

It should be noted, however, that the climate thresholds derived from our RF model should not be interpreted as fixed physiological limits. These thresholds reflect modeled responses based on regional data (2001–2019) and may vary with cultivar, management practices, and interannual climatic conditions. WW and OSR varieties grown in Bavaria have differing phenological traits and climate adaptation strategies, which likely influence their observed sensitivity. Future studies that incorporate cultivar-specific data and more detailed phenological modeling could provide further insight into the robustness and generalizability of these thresholds.

### 4.4 Role of topography and soil in biomass patterns

The study results highlight the critical influence of topography and soil on spatial patterns of crop biomass, reinforcing prior evidence that these factors shape yield stability in heterogeneous agricultural landscapes. Across Bavaria, terrain complexity varies substantially, from the high-elevation Alps and Pre-Alps to the gently rolling Tertiary Hills Region and the fertile plains of Franconia, each presenting distinct constraints and opportunities for crop growth. Steep slopes (>3°–8°) significantly limit biomass production for both WW and OSR. These areas are often associated with shallower soils, higher erosion risks, and reduced mechanization efficiency: factors that can constrain water and nutrient availability and ultimately limit crop growth potential (Chen et al., 2022; Lann et al., 2024). In upland regions like the Alps, Pre-Alps, and East-Bavarian Mid-Range Mountains, such topographic constraints likely intensify the impact of climatic stressors by reducing thermal accumulation and shortening effective growing periods.

Conversely, the Tertiary Hills Region and Franconia, characterized by moderate slopes and deeper, fertile soils, consistently supported higher biomass. This corresponds with the study of Pennock et al. (1992), showing that gently sloped, lowland areas accumulate water and nutrients more effectively and offer more stable yield conditions. Such zones typically exhibit less interannual yield volatility due to favorable soil texture, organic content, and moisture retention.

Biomass increases sharply with soil potential beyond values of 50–60 and plateaus near 75, indicating a strong positive relationship between soil quality and productivity for both crops, as shown in the PDPs for WW and OSR (**Figures 10C** and **11C**). These thresholds align with previous research demonstrating that high-yield zones correlate with favorable edaphic traits, where soil quality can compensate for moderate climatic stress (Van Wart et al., 2013).

The study results underscore the need to consider the terrain and edaphic variability when modeling biomass at fine spatial scales. While climate sets the baseline for productivity, the realization of yield potential is strongly modulated by topographic and soil conditions. These factors are especially variable across Bavaria’s diverse agricultural zones. Integrating these variables into machine learning models enhances predictive accuracy and provides a more comprehensive understanding of local yield determinants.

The study results underscore the importance of integrating both climatic and non-climatic variables when modeling biomass at fine spatial scales. Climatic factors (e.g., solar radiation, temperature, precipitation) define the fundamental biophysical limits for photosynthesis and growth, but the actual realization of yield potential is strongly modulated by soil and topographic conditions, which influence water availability, drainage, and nutrient uptake. These factors vary substantially across Bavaria’s heterogeneous landscapes and are critical for explaining local deviations in biomass. Prior studies have also emphasized the role of soil and terrain in shaping yield outcomes under comparable climatic settings (e.g (Folberth et al., 2016; Grassini et al., 2015)).

### 4.5 Limitations and outlook

This study offers valuable insights into spatial patterns of crop biomass by integrating a semi-empirical LUE model with a data-driven RF approach. While the framework performed robustly across Bavaria’s diverse agricultural regions, several limitations should be acknowledged. First, hexagons with less than 5 hectares of WW or OSR were excluded to reduce noise from sparsely cultivated areas. Although this improved model stability, it may have limited the representation of smallholder and fragmented fields, especially in regions like the EBMRM and the Alps. Including smaller fields with improved classification methods could enhance spatial coverage in future applications.

Second, the model did not include management practices such as crop rotation, fertilization, or tillage, which are known to influence biomass production and its variability. Incorporating such data, where available, could increase model accuracy and relevance for precision agriculture. Third, while LUE-derived biomass maps provide a physiologically meaningful response variable, they do not directly reflect harvested yields and are subject to assumptions related to crop-specific stress responses. This distinction is crucial, as end-users such as farmers and policymakers are primarily concerned with actual yields. Several studies highlight that the biomass–yield relationship can decouple under extreme climatic conditions. For instance, excessive rainfall may not substantially reduce biomass but can increase the risk of fungal diseases, lodging, or incomplete grain filling, ultimately reducing harvestable yield (Trnka et al., 2014). Similarly, heat stress during reproductive phases can drastically reduce grain or seed formation despite sufficient vegetative biomass (Zhao et al., 2017). Therefore, further calibration using *in-situ* biomass and yield observations, especially under extreme weather conditions, could enhance the reliability of biomass-based yield proxies. This could improve the applicability of such models in climate-resilient agricultural planning. Additionally, crop-specific differences in the distribution of predicted biomass values suggest that model refinements, particularly for low-biomass WW fields, could further enhance predictive accuracy. While this study focused on RF regression models for predictive modeling, future research could explore how other machine learning algorithms compare in terms of accuracy, interpretability, and computational cost, especially in data-rich agricultural systems.

Despite these limitations, the modeling framework demonstrates potential for transferability. This stems from the simplicity of using the LUE model to estimate biomass from remote sensing data to different crop types and the flexibility of integrating its outputs with RF modeling. Additionally, the framework relies solely on available climate inputs (e.g., ERA-Interim ERA-Interim from the European Centre for Medium-Range Weather Forecasts [ECMWF]), land use and land cover maps, and incorporates spatially aggregated predictors, such as climate, soil, topography, and landscape structure, wherever such data are available. This makes the approach adaptable to other crops and agroecosystems, particularly in temperate or topographically complex regions.

## 5 Conclusions

This study presents a spatially explicit framework for understanding the environmental factors influencing biomass production in winter wheat and oilseed rape across Bavaria, Germany. By combining remote sensing–derived biomass with a machine learning approach and multi-source environmental predictors within a hexagon-based spatial structure, the study captures both spatial variability and interannual climatic fluctuations from 2001 to 2019.

The findings show that integrating remote sensing with environmental variables, such as climate, landscape structure, topography, and soil potential, offers a robust approach for characterizing regional biomass patterns. Landscape configuration emerged as an important driver, with moderate levels of land-cover diversity and small woody feature coverage associated with increased biomass. However, excessive landscape variability, especially in land-use diversity, corresponded with biomass reductions, indicating potential trade-offs between complexity and productivity.

Climatic factors also played a key role, with crop-specific thresholds evident in response to temperature, solar radiation, and precipitation variability. Notably, winter wheat showed higher sensitivity to interannual climatic fluctuations, while oilseed rape displayed resilience in more stable early-season conditions.

By incorporating both mean and variability metrics of environmental drivers, the modeling framework effectively explained spatial patterns in crop biomass across ecologically diverse regions. This emphasizes the importance of accounting not only for average growing conditions but also for the stability of those conditions over time.

The approach developed here is scalable and transferable, particularly in regions where remote sensing and environmental data are accessible. Future research should expand this framework to additional crop types, integrate temporal land-use dynamics, and incorporate farm-level management practices to better capture decision-driven variability. Doing so will help enhance the precision of agricultural planning and support the development of resilient, climate-adaptive farming systems under ongoing environmental change.

## Data Availability

The datasets presented in this study can be found in online repositories. The names of the repository/repositories and accession number(s) can be found in the article/**Supplementary Material**.
