# Supplementary material for: Landscape structure, climate variability, and soil quality shape crop biomass patterns in agricultural ecosystems of Bavaria
Source: Front Plant Sci. 2025 Aug 7;16:1630087. doi: 10.3389/fpls.2025.1630087 (PMC12367677; doi:10.3389/fpls.2025.1630087)
Supplement: Supplementary file 1 [file DataSheet1.docx]

Supplementary Data

Landscape Structure, Climate Variability, and Soil Quality Shape Crop Biomass Patterns in Agricultural Ecosystems of Bavaria

Maninder Singh Dhillon^1*^, Thomas Koellner^2^, Sarah Asam^3^, Jakob Bogenreuther^2^, Stefan Dech^1,3^, Ursula Gessner^3^, Daniel Gruschwitz^1^, Sylvia Helena Annuth^2^, Tanja Kraus^3^, Thomas Rummler^4^, Christian Schaefer^1^, Sarah Schönbrodt-Stitt^1^, Ingolf Steffan-Dewenter^5^, Martina Wilde^1,6^, Tobias Ullmann^1^

^1^  Department of Remote Sensing, Institute of Geography and Geology, University of Würzburg, 97074 Würzburg, Germany

^2^ Department of Ecological Services, Faculty of Biology, Chemistry and Earth Sciences, Bayreuth Center of Ecology and Environmental Research (BayCEER), University of Bayreuth, 95447, Bayreuth, Germany

^3^ German Remote Sensing Data Center (Deutsches Fernerkundungsdatenzentrum, DFD), German Aerospace Center (Deutsches Zentrum für Luft- und Raumfahrt, DLR), 82234 Wessling, Germany

^4^  Department of Applied Computer Science, Institute of Geography, University of Augsburg, 86159 Augsburg, Germany

^5^  Department of Animal Ecology and Tropical Biology, University of Würzburg, 97074 Würzburg, Germany

^6^  Department of Physical Geography and Soil Science, Institute of Geography and Geology, University of Würzburg, 97074 Würzburg, Germany

**^*^** Correspondence: maninder.dhillon@uni-wuerzburg.de

**Appendix A**

**Figure A1:** Spatial distribution of key climate predictors used for biomass modeling of winter wheat (WW) across Bavaria from 2001 to 2019. **(A)** Mean growing-season temperature (°C), **(B)** standard deviation (SD) of growing-season temperature (°C), **(C)** mean growing-season solar radiation (W m⁻² day⁻¹), **(D)** SD of growing-season solar radiation (W m⁻² day⁻¹), **(E)** mean growing-season precipitation (mm day⁻¹), and **(F)** SD of growing-season precipitation (mm day⁻¹). Maps show hexagon-based aggregations, where warmer colors (red, orange) represent higher temperatures and solar radiation, and cooler colors (blue) indicate higher precipitation levels. Greater SD values reflect higher interannual climatic variability during the crop's growing season.

**Figure A2:** Spatial distribution of key climate predictors used for biomass modeling of oilseed rape (OSR) across Bavaria from 2001 to 2019. **(A)** Mean growing-season temperature (°C), **(B)** standard deviation (SD) of growing-season temperature (°C), **(C)** mean growing-season solar radiation (W m⁻² day⁻¹), **(D)** SD of growing-season solar radiation (W m⁻² day⁻¹), **(E)** mean growing-season precipitation (mm day⁻¹), and **(F)** SD of growing-season precipitation (mm day⁻¹). Maps show hexagon-based aggregations, where warmer colors (red, orange) represent higher temperatures and solar radiation, and cooler colors (blue) indicate higher precipitation levels. Greater SD values reflect higher interannual climatic variability during the crop's growing season.

**Figure A3**: Spatial distribution of topographic and soil predictors used for biomass modeling of winter wheat (WW) and oilseed rape (OSR) across Bavaria. **(A)** Mean elevation (m a.s.l.), **(B)** standard deviation (SD) of elevation (m a.s.l.), **(C)** mean slope angle (°), **(D)** SD of slope (°), **(E)** mean soil potential (%), and **(F)** SD of soil potential (%). Predictors were aggregated to a 5 km² hexagon grid based on crop-specific field boundaries. Color gradients reflect the magnitude of each variable per hexagon, with darker shades indicating higher values. SD maps represent intra-hexagon variability.

**Figure A4**: Spatial visualization of key landscape structure predictors for biomass modeling across Bavaria. **(A)** Map showing the distribution of small woody features (SWFs) (%) per hexagon, with greener tones indicating higher SWF density. A zoomed-in hexagon highlights an area with a high SWF concentration. **(B)** Map displaying the mean Shannon Diversity Index (SHDI) per hexagon, where bluish tones represent higher overall land cover diversity. **(C)** Map showing the standard deviation (SD) of SHDI per hexagon, reflecting intra-hexagon landscape variability. Moderate SD values indicate areas where some parts are highly diverse, while other areas remain uniform (dominated by forest or agricultural fields).

**Appendix B**

The datasets supporting the findings of this study are publicly available via Zenodo at: https://doi.org/10.5281/zenodo.15389404. This includes spatial and spatio-temporal predictor layers, biomass outputs, and SHAP/PDP results for winter wheat and oilseed rape from 2001 to 2019 across Bavaria.
