# Supplementary figures and images for: Landscape structure, climate variability, and soil quality shape crop biomass patterns in agricultural ecosystems of Bavaria
Source: Front Plant Sci. 2025 Aug 7;16:1630087. doi: 10.3389/fpls.2025.1630087 (PMC12367677; doi:10.3389/fpls.2025.1630087)

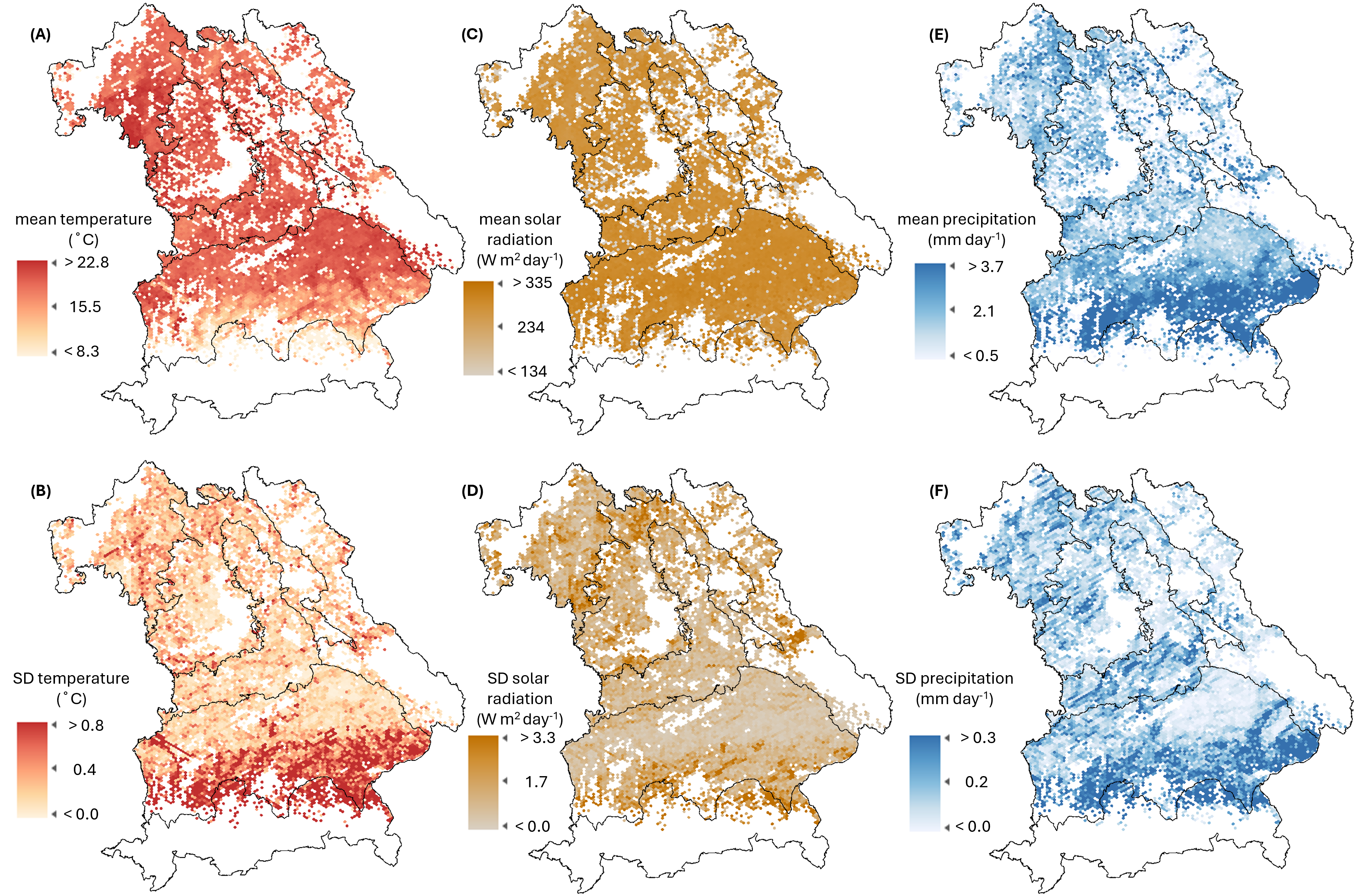

Supplement: Supplementary file 2 [file Image1.tif]

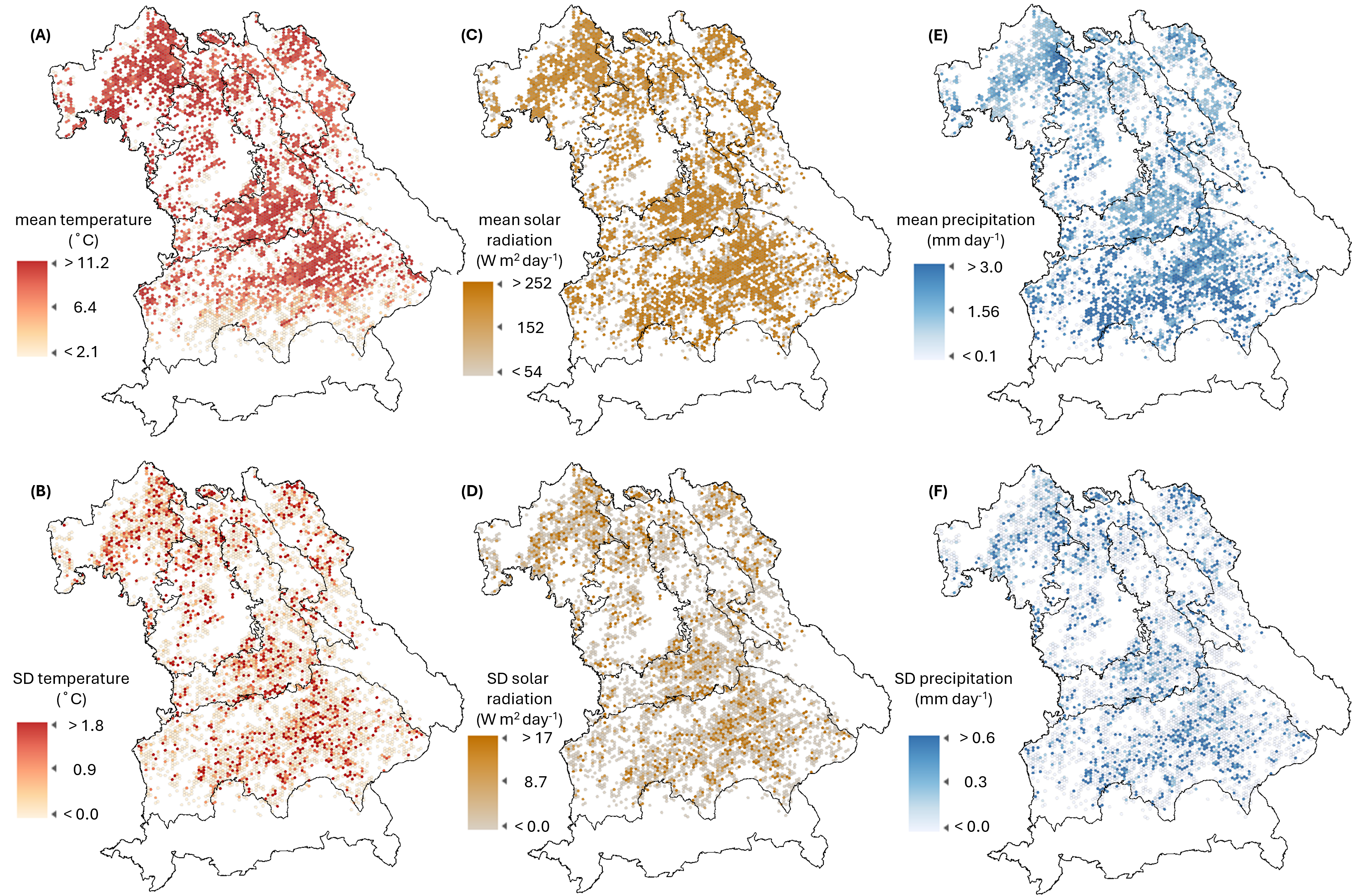

Supplement: Supplementary file 3 [file Image2.tif]

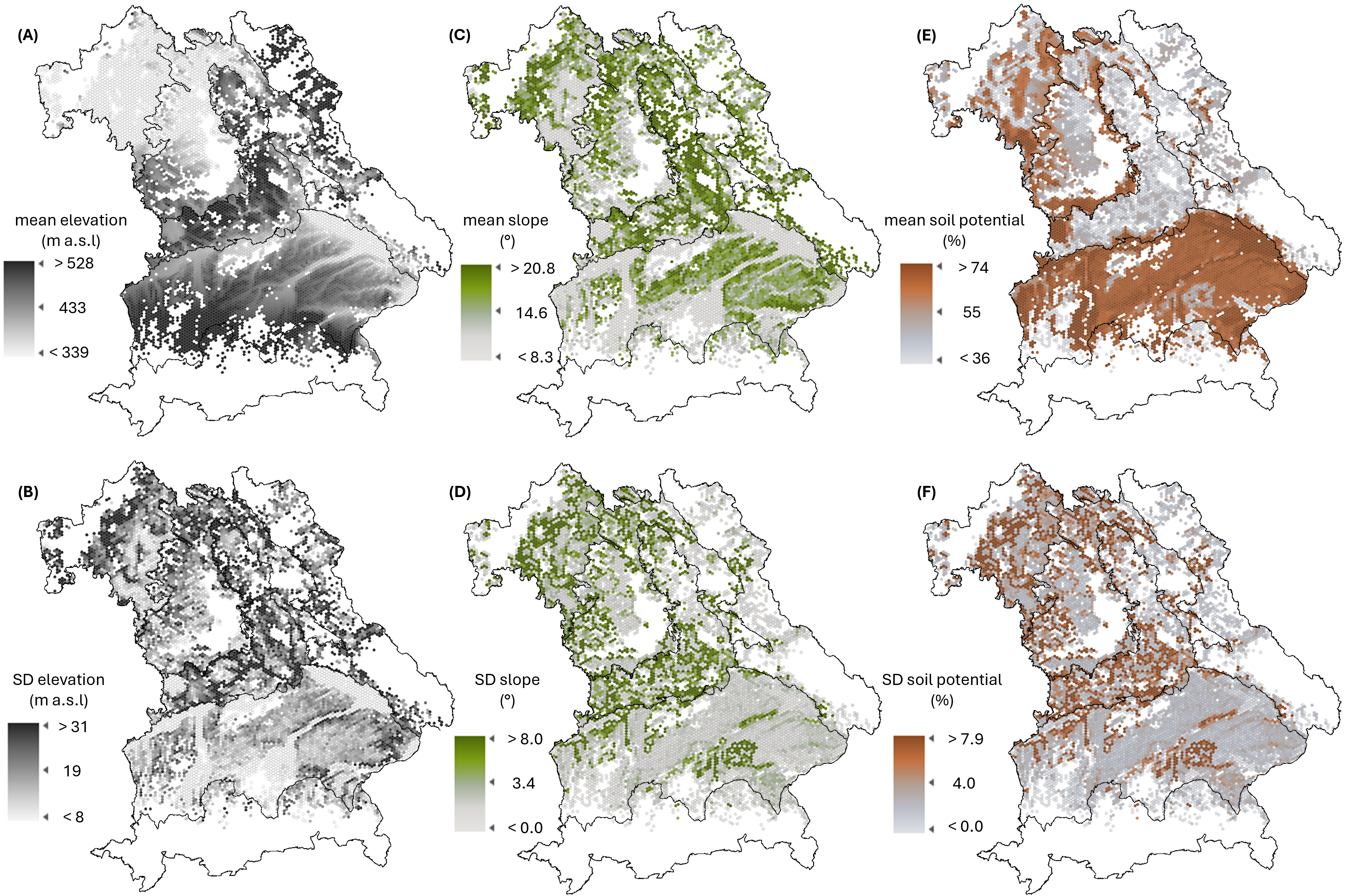

Supplement: Supplementary file 4 [file Image3.tif]

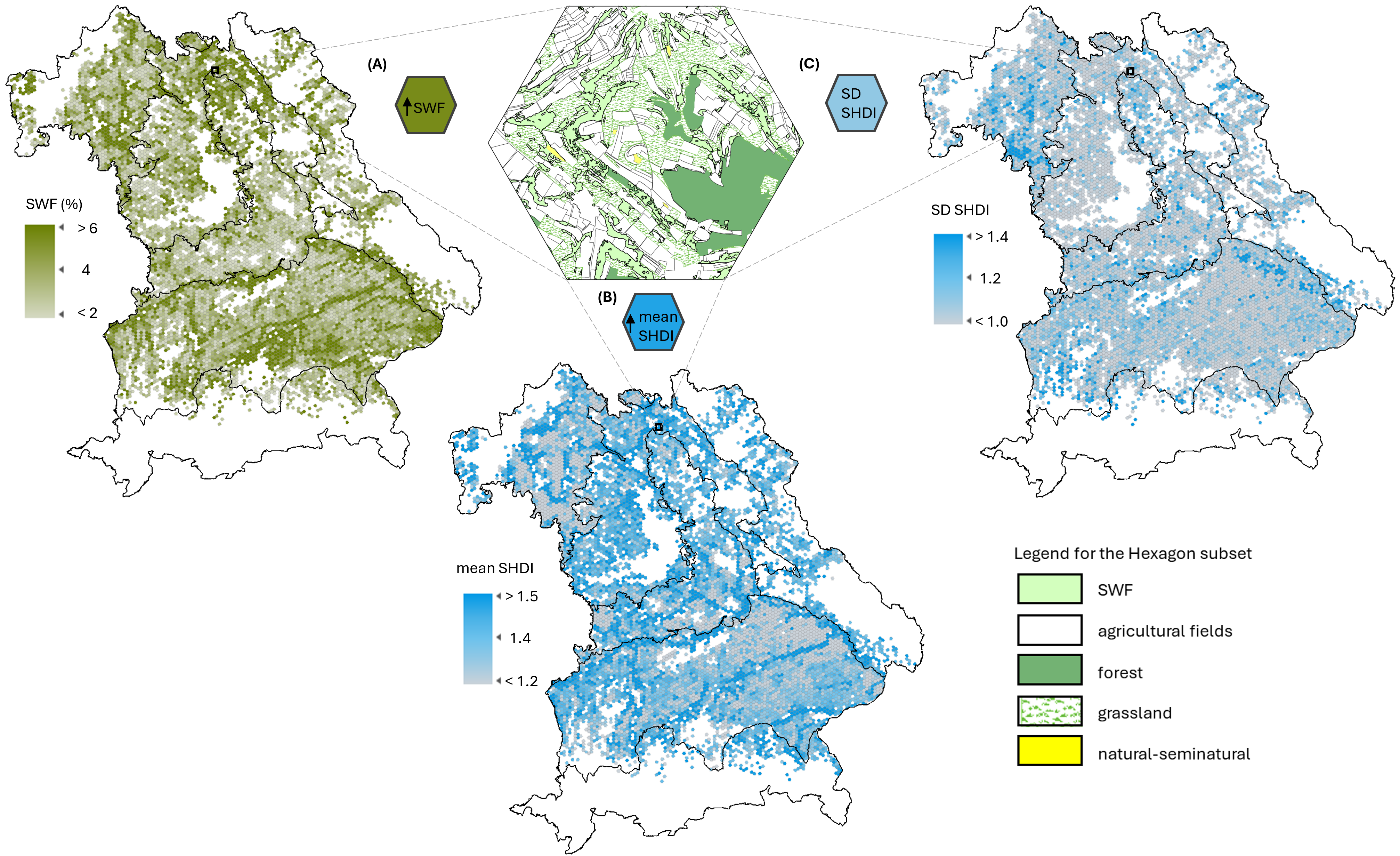

Supplement: Supplementary file 5 [file Image4.tif]
